# EPI-DynFusion: enhancer-promoter interaction prediction model based on sequence features and dynamic fusion mechanisms

**DOI:** 10.3389/fgene.2025.1614222

**Published:** 2025-07-23

**Authors:** Ao Zhang, Jianhua Jia, Mingwei Sun, Xin Wei

**Affiliations:** ^1^School of Information Engineering, Jingdezhen Ceramic University, Jingdezhen, China; ^2^Business School, Jiangxi Institute of Fashion Technology, Nanchang, China

**Keywords:** EPI prediction, dynamic feature fusion, transformer, CBAM, deep learning

## Abstract

**Introduction:**

Enhancer–promoter interactions (EPIs) play a vital role in the regulation of gene expression. Although traditional wet-lab methods provide valuable insights into EPIs, they are often constrained by high costs and limited scalability. As a result, the development of efficient computational models has become essential. However, many current deep learning and machine learning approaches utilize simplistic feature fusion strategies, such as direct averaging or concatenation, which fail to effectively model complex relationships and dynamic importance across features. This often results in suboptimal performance in challenging biological contexts.

**Methods:**

To address these limitations, we propose a deep learning model named EPI-DynFusion. This model begins by encoding DNA sequences using pre-trained DNA embeddings and extracting local features through convolutional neural networks (CNNs). It then integrates a Transformer and Bidirectional Gated Recurrent Unit (BiGRU) architecture with a Dynamic Feature Fusion mechanism to adaptively learn deep dependencies among features. Furthermore, we incorporate the Convolutional Block Attention Module (CBAM) to enhance the model’s ability to focus on informative regions. Based on this core architecture, we develop two variants: EPI-DynFusion-gen, a general model, and EPI-DynFusion-best, a fine-tuned version for cell line–specific data.

**Results:**

We evaluated the performance of our models across six benchmark cell lines. The average area under the receiver operating characteristic curve (AUROC) scores achieved by the specific, generic, and best models were 94.8%, 95.0%, and 96.2%, respectively. The average area under the precision-recall curve (AUPR) scores were 81.2%, 71.1%, and 83.3%, respectively, demonstrating the superior performance of the fine-tuned model in the precision-recall space. These results confirm that the proposed fusion strategies and attention mechanisms contribute to significant improvements in performance.

**Discussion:**

In conclusion, EPI-DynFusion presents a robust and scalable framework for predicting enhancer–promoter interactions solely based on DNA sequence information. By addressing the limitations of conventional fusion techniques and incorporating attention mechanisms alongside sequence modeling, our method achieves state-of-the-art performance while enhancing the interpretability and generalizability of enhancer–promoter interaction prediction tasks.

## 1 Introduction

Enhancers and promoters are essential components in the regulation of gene expression. Promoters are situated upstream of genes and directly control the binding of RNA polymerase II (Hahn, 2004) and the initiation of transcription, while enhancers function to increase the expression of target genes by binding to transcription factors (Spitz and Furlong, 2012). In recent years, enhancer-promoter interactions (EPIs) have emerged as a significant focus in gene control research. EPIs refer to the physical contact and functional collaboration between enhancers and promoters, typically achieved through three-dimensional structural changes in chromatin, such as the formation of chromatin loops (Schoenfelder and Fraser, 2019). EPIs not only facilitate the recruitment of transcription factors and coactivators but also regulate the spatial and temporal expression of genes, exhibiting specific tissue or cellular specificity (Zhang et al., 2013). The dynamics and plasticity of EPIs play vital roles in biological development, disease processes, and environmental responses. Consequently, identifying EPIs is essential for unraveling complex gene regulatory networks and holds great significance for gene therapy and studies of disease mechanisms.

In recent years, both biological methods and computational techniques for identifying enhancer-promoter interactions (EPIs) have advanced significantly. Chromatin conformation capture techniques, such as 3C, Hi-C, and ChIA-PET (Dekker et al., 2002; Fullwood et al., 2009; Lieberman-Aiden et al., 2009), are commonly employed experimental tools that reveal the three-dimensional structure of chromatin and determine the physical contacts between distant DNA fragments. However, these methods have notable limitations. First, they typically require a large number of samples, leading to high experimental costs and limited resolution, which can hinder the accurate capture of specific EPIs (Mifsud et al., 2015). Second, traditional techniques often provide only a static view of chromatin conformation, failing to fully represent the dynamic regulation of gene expression (Bonev and Cavalli, 2016). Additionally, these methods are highly dependent on chromatin status and cell type, resulting in a lack of generalizability (Rao et al., 2014; Stadhouders et al., 2019).

Meanwhile, machine learning techniques have demonstrated significant advantages in identifying EPIs. Relevant studies are primarily categorized into two main groups: genomic data-based approaches and sequence information-based approaches. Among the genomic data-based methods, the TargetFinder model developed by Whalen et al. (2016). Integrates multiple genomic features, such as transcription factor ChIP-seq, histone markers, and DNA methylation, to predict EPIs. This model relies on a comprehensive understanding of specific genomic features and has achieved favorable results. In contrast, sequence information-based approaches identify EPIs by directly extracting features from DNA sequences. The PEP-WORD model proposed by Yang et al. (2017). Employs word embedding techniques alongside boosted tree integration algorithms, while the EPIANN model designed by Weiguang et al. (2017). Incorporates an attention mechanism and decodes positional features. Additionally, the SPEID model developed by Shashank et al. (2019). Combines long short-term memory (LSTM) (Sepp and Jürgen, 1997) networks with CNN, the SIMCNN model proposed by Zhuang et al. (2019). Enhances prediction efficiency by integrating CNNs with transfer learning. The EPIVAN model proposed by Hong et al. (2020). Utilizes pre-trained DNA vectors, such as dna2vec, to encode enhancers and promoters. It combines CNN and gated recurrent units (GRU) to extract both local and global features, ultimately enhancing the contribution of key features through an attention mechanism. In contrast, the EPI-DLMH model developed by Min et al. (2021). Integrates a two-layer CNN with matching heuristics to predict interactions between enhancers and promoters. Both models effectively leverage deep learning technology to extract critical features from DNA sequences, thereby providing robust support for the accurate prediction of enhancer-promoter interactions.

Recently, models based on the Transformer architecture have demonstrated significant potential in predicting EPIs. Notable examples of such models include EPI-Mind, proposed by Ni et al. (2022). And EPI-Trans, introduced by Ahmed et al. (2024). EPI-Mind integrates CNN and Transformer architectures by initially employing CNN for feature extraction from sequences, followed by inputting the extracted features into the Transformer module for further processing. This two-step approach enables the model to capture both local sequence patterns and long-range dependencies. Similarly, EPI-Trans also combines CNN and Transformer, but it first processes enhancer and promoter sequences separately using CNN (Yann et al., 2015). The extracted features are then merged and fed into the Transformer module, allowing the model to learn joint features of enhancers and promoters and effectively capture their interactions. Overall, these models leverage the strengths of CNN in localized feature extraction alongside the Transformer’s capability to handle long sequences and capture long-distance dependencies.

Building on this, several new approaches have recently emerged in deep learning-based enhancer-promoter interaction prediction models. For instance, the EPIHC model proposed by Liu et al. (2021). Utilizes genomic data to generate heatmaps of enhancer-promoter interactions, thereby revealing potential regulatory relationships. EPIHC enhances prediction by integrating comprehensive genomic information, including transcription factor binding sites, epigenetic markers, and transcription factor activities. The visualization of heatmaps intuitively illustrates potential interaction patterns between enhancers and promoters. Additionally, the TF-EPI model, also proposed by Liu et al. (2024), emphasizes the prediction of EPIs through the role of transcription factors. This model primarily analyzes transcription factor binding sites within DNA sequences and employs machine learning algorithms to infer interactions between enhancers and promoters. Meanwhile, the RAEPI (Zhang et al., 2025) model effectively captures the complex and dynamic regulatory relationships between enhancers and promoters from the core perspective of ‘regulatory activity.’ It considers gene expression levels, epigenetic modifications, and transcription factor signals using deep neural networks. RAEPI underscores the synergistic nature of the regulatory activities of enhancers and promoters. By incorporating biologically relevant features, it further enhances the model’s predictive ability within the context of complex gene regulation. Collectively, these models highlight the critical roles of transcription factors and epistatic regulatory information in gene regulatory networks, providing more accurate prediction results through various methodologies. Most of the existing models suffer from relatively fixed feature fusion strategies. Most existing models are hindered by relatively fixed feature fusion strategies, a lack of sensitivity to dynamic interactions among sequences, and limited performance in addressing complex nonlinear relationships. In particular, static feature fusion methods struggle to capture fine-grained dynamic interaction patterns, especially when the importance of features fluctuates with context or when interactions are weak. This limitation adversely affects the predictive accuracy and generalization ability of the models in complex biological scenarios.

To address the limitations of existing feature fusion methods, this study proposes a novel deep learning model for dynamic feature fusion, named EPI-DynFusion, which is based on the Transformer architecture. Unlike traditional fixed-weight fusion methods, we design an attention-guided dynamic feature fusion mechanism that can adaptively adjust the fusion ratios of features at different levels by learning a dynamic weight matrix based on specific sequence characteristics. Specifically, the model first uses Convolutional Neural Networks to extract preliminary features, and then combines the Transformer architecture with a bidirectional gating unit to capture global features. During the feature fusion stage, the model achieves more precise feature representation through adaptive weighting of different features using a dynamic weight matrix. Finally, we incorporate CBAM to further enhance sequence feature extraction. Experimental results show that this dynamic feature fusion strategy improves model performance to a certain extent, making EPI-DynFusion an advanced tool for predicting enhancer-promoter interactions.

## 2 Methods

### 2.1 Data

In this study, we utilized an enhancer-promoter interaction dataset from TargetFinder (Whalen et al., 2016) that encompasses six human cell lines: GM12878, HUVEC, HeLa-S3, IMR90, K562, and NHEK. The regulatory elements within this dataset were identified by integrating annotation information from ENCODE and Roadmap Epigenomics. Specifically, promoter regions were defined as sequence fragments containing transcription start sites (TSS) exhibiting active transcriptional activity (average FPKM >0.3, non-repeatable discovery rate <0.1). Enhancer regions were annotated based on characteristic chromatin modifications and were excluded if they were located within 10 kb of the nearest promoter. Interactions between enhancers and promoters were determined using high-resolution genome-wide Hi-C data (FDR <10%), where significant interactions served as positive samples and non-interacting pairs with similar genomic distances were designated as negative samples (Rao et al., 2014). We selected this dataset due to its rigorous experimental validation and its widespread use as a benchmark for assessing the performance of enhancer-promoter interaction prediction models.

In the dataset for each cell line, the ratio of positive samples to negative samples is approximately 1:20, and in most instances, enhancers do not interact with promoters. Some negative samples may lack the characteristics of non-interacting pairs, resulting in an underrepresentation of samples in the training set. Furthermore, imbalanced data can adversely affect model performance, particularly in supervised deep learning models, which may lead the model to excessively focus on the dominant categories (Chawla et al., 2004; He and Garcia, 2009). This overemphasis can diminish the prediction accuracy for less prevalent categories and introduce negative bias into the model. To mitigate the issue of data imbalance, we augment the number of positive samples by up to 20 times using data augmentation techniques prior to training, thereby ensuring a balanced ratio of positive to negative samples in the dataset (Shorten and Khoshgoftaar, 2019). Specifically, the sequence lengths for the enhancer and promoter were 3000 bp and 2000 bp, respectively. We expanded the regions by sliding a fixed-size window either to the left or right of the DNA sequence, while ensuring that the expanded regions encompassed the majority of the functional elements (Zhou and Troyanskaya, 2015). Our sliding window strategy ensures that each augmented sample contains unique combinations of sequence regions, as even minor changes in base pairs can introduce certain biological differences. This approach, which is based on the data enhancement technique described in (Weiguang et al., 2017), effectively balances the sample categories in the training set, resulting in a balanced dataset as illustrated in Table 1.

**TABLE 1:** Number of positive, enhanced positive and negative samples in six cell lines.

Cell lines	Training dataset			Test dataset	
	Pos samples	Aug.Pos samples	Neg samples	Pos samples	Neg samples
GM12878	1,902	38,040	37,980	211	4,220
HUVEC	1,372	31,320	31,320	174	3,480
HeLa-S3	1,566	27,440	27,360	152	3,040
IMR90	1,129	22,580	22,500	125	2,500
K562	1,780	35,600	35,550	197	3,950
NHEK	1,162	23,240	23,040	129	2,560
Total	8,911	178,220	177,750	988	19,750

### 2.2 Model structure

**FIGURE 1:**
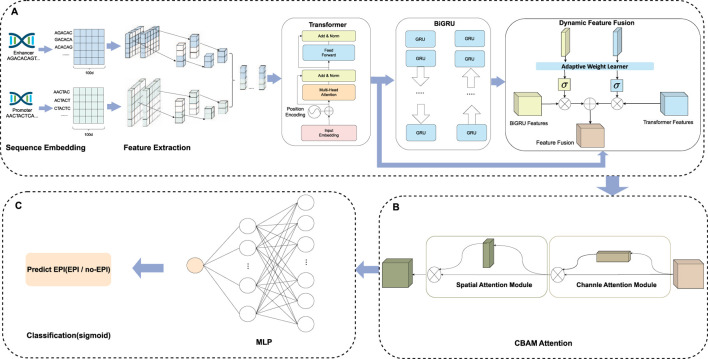
The EPI-DynFusion model structure. **(A)** Feature extraction and dynamic feature fusion part of EPI-DynFusion: We propose EPI-DynFusion, a breakthrough framework addressing the challenge of effectively integrating multi-level features in traditional enhancer-promoter interaction prediction methods. Our model innovatively develops a dynamic feature fusion-driven deep learning framework. EPI-DynFusion (where “EPI” represents enhancer-promoter interaction, “Dyn” emphasizes dynamic feature extraction, and “Fusion” reflects multi-dimensional feature integration) introduces three key technologies: Utilizing dna2vec embedding to effectively represent DNA sequence semantic features; Pioneering a dual-encoding architecture combining Transformer and BiGRU to enhance sequence modeling capabilities; Designing an innovative dynamic feature fusion mechanism to adaptively integrate multi-level feature representations improves the model’s prediction performance for enhancer-promoter interactions to a certain extent. **(B)** CBAM attention part of EPI-DynFusion: Channel attention and spatial attention mechanisms are incorporated to further evaluate the importance of high-level features, with the two attention mechanisms being concatenated in a sequential manner. **(C)** Prediction Module. The final prediction probabilities are obtained through a fully connected neural network that serves as the classification layer.

**FIGURE 2:**
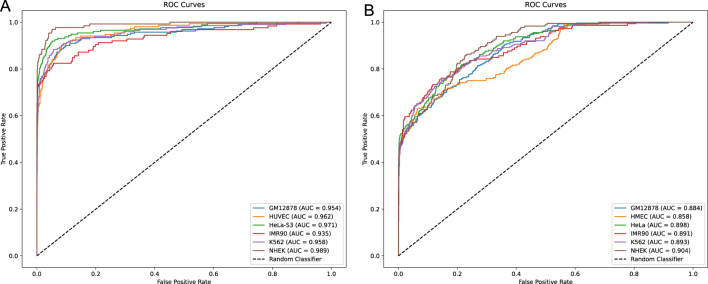
ROC curves for independent testing. **(A)** ROC curve obtained from the independent test of the TargetFinder dataset. **(B)** ROC curve obtained from the independent test of the BENGI dataset. In both cases, all models’ ROC curves lie above the diagonal, indicating that they outperform the random classifier. Compared to the more similar model performance in **(A)**, **(B)** demonstrates a more pronounced performance difference, with certain models achieving better performance and higher classification accuracy.

In this study, we developed a network framework named EPI-DynFusion for the automated detection of EPIs using DNA sequences. The EPI-DynFusion framework consists of three main components: sequence embedding, the EPI-DynFusion model framework, and EPI prediction, as illustrated in Figure 1. Initially, enhancer and promoter sequences are input into the model, generating feature matrix embeddings using a pre-trained dna2vec method. Local features are then extracted through a convolutional layer, followed by the application of a Transformer encoder to capture global dependencies. Unlike traditional fixed-weight fusion approaches, we propose a novel dynamic feature fusion mechanism that adaptively calibrates feature weights based on the contextual information of each sequence. Specifically, our model learns a dynamic weight matrix that intelligently adjusts the fusion ratios between Transformer and BIGRU features, enabling sequence-specific feature emphasis. This adaptive fusion is further enhanced by integrating the Convolutional Block Attention Module (CBAM) (Woo et al., 2018), which refines both channel and spatial feature representations. Through this dynamic fusion strategy, our model can automatically identify and emphasize the most discriminative features for each specific sequence pair, leading to more accurate EPI prediction results through the prediction layer. Figure 2A presents the ROC plot derived from the independent test of the dataset constructed by TargetFinder, while Figure 2B displays the ROC plot from the independent test of the dataset developed by BENGI (Liu et al., 2024). The results show that our proposed EPI-DynFusion model has good robustness. In the following sections, we will elaborate on the proposed framework in detail.

#### 2.2.1 Sequence embedding

In this study, we employed a sequence-embedding method to analyze long DNA sequences, aiming to address the limitations associated with traditional coding approaches. We initially applied the k-mer characterization method to partition promoter and enhancer sequences using a sliding window of length k and a step size of s (Yang et al., 2017). Specifically, the k-mer method segments the gene sequence into multiple short k-base pair segments. Previous research has demonstrated that the computational efficiency and information complexity of the vectors are optimal when k is set to 6. Consequently, we also set k to 6 and the step size s to 1 in this study.

In the process of sequence embedding, we compare two distinct methods: One-hot encoding and dna2vec (Patrick, 2017; Zou et al., 2019). While One-hot encoding is relatively straightforward to implement and provides an intuitive vector representation, it suffers from the issue of dimensionality catastrophe. For a k-mer with k = 6, One-hot encoding generates vectors with a dimensionality of 4^6 = 4,096. This high-dimensional representation not only significantly increases computational complexity but also heightens the sparsity of the data matrix, adversely affecting the model’s ability to analyze biological sequences. Furthermore, One-hot encoding is limited in its capacity to capture correlations between bases, which restricts the model’s ability to comprehend the underlying contextual information in the sequence. To address these challenges, we employ the dna2vec method for sequence embedding in this study. Dna2vec is an innovative word2vec-based approach that produces high-quality, low-dimensional continuous vectors to represent k-mer fragments. It is trained using a shallow two-layer neural network on large-scale DNA sequences (e.g., hg38 chromosomes 1 through 22 of the human genome) to learn embedding representations of k-mer fragments. In this study, we utilize 6-mer length sequences for embedding, ensuring consistency of the k-mer fragments within the same embedding vector space. This fixed length of 6-mer allows the model to capture pattern information in gene sequences more efficiently.

**FIGURE 3:**
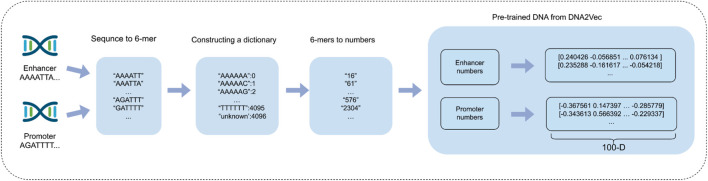
Enhancer and promoter sequence embedding using DNA2Vec pre-training method.

In this specific experimental setup, enhancer sequences and promoter sequences are represented as matrices with dimensions of 3,000 × 100 and 2,000 × 100, respectively. This embedding approach enables each DNA sequence to be efficiently represented as a vector that retains rich information and low-dimensional features, thereby assisting downstream deep learning models in more effectively capturing potential characteristics of biological sequences. By employing the dna2vec embedding method, we successfully tackle the challenges of dimensionality catastrophe and sparse matrix issues commonly associated with traditional one-hot encoding. This advancement effectively enhances computational efficiency while preserving the contextual relevance of biological sequences, ultimately leading to improvements in the accuracy and efficiency of gene function prediction. Figure 3 shows the process of using the dna2vec pre-training method in detail.

#### 2.2.2 Feature extraction

To enhance feature extraction efficiency, we employ two independent CNN networks: one dedicated to enhancers and the other to promoters. Each CNN consists of a one-dimensional convolutional layer followed by a maximum pooling layer. The one-dimensional convolutional layer enables the model to learn local features from the input sequence, while the maximum pooling layer subsequently reduces the dimensions of the features, simplifying further computations. After the convolution operation, we apply the ReLU activation function to improve the model’s capacity to capture nonlinear features. By utilizing these two independent CNN networks, the model effectively captures the unique characteristics of enhancer and promoter sequences, which are then integrated through a merging layer. To mitigate the risk of overfitting, we incorporate batch normalization and a dropout layer following the merging layer, thereby enhancing the model’s generalization ability.

##### 2.2.2.1 Transformer

To extract high-level global features, we employ the Transformer technique originally proposed by Vaswani et al. (2017). The distinctive aspect of the Transformer mechanism is its capability to capture positional information, thereby facilitating the automatic acquisition of additional information during the feature extraction process. Figure 1 illustrates the Transformer mechanism, which comprises four core modules: positional encoding, multi-head attention, position-wise feed-forward networks, and residual connections with layer normalization. For further details regarding these modules, please refer to the work of Vaswani et al.

During the network design process, we focused on the interrelated constraints between the Transformer model and the two-layer convolutional neural network concerning hyperparameter settings. First, in the design of the one-dimensional convolutional layer, the number of selected filters must correspond to the dimensionality of the Transformer model. Specifically, the addition and normalization modules (add and norm) within the Transformer architecture are positioned between the input layer and the multi-head attention output, necessitating that the number of filters aligns with the model’s dimensionality. Additionally, the multi-head attention module in the Transformer imposes specific requirements on the model’s dimensionality; it must be an integer multiple of the number of attention heads to ensure effective feature delineation.

In addition, the filter sizes of the 1D convolutional layers in both the enhancer and the initiator are set to 80 and 61, respectively, with a step size of 1. The pooling sizes of the 1D max pooling layers are set to 15 and 10, respectively, with a step size that aligns with the pooling size. For the model dimensions of the Transformer and the number of filters in the convolutional layer, we have uniformly established these parameters at 100. The Transformer module comprises one coding stack, ten multi-head attention heads, and a feedforward layer containing 256 hidden units. With these configurations, we successfully achieved a tight integration of the CNN and Transformer modules, thereby equipping the network with the capability to effectively extract both global and local features, which subsequently enhances the overall performance of the model.

##### 2.2.2.2 BiGRU

**FIGURE 4:**
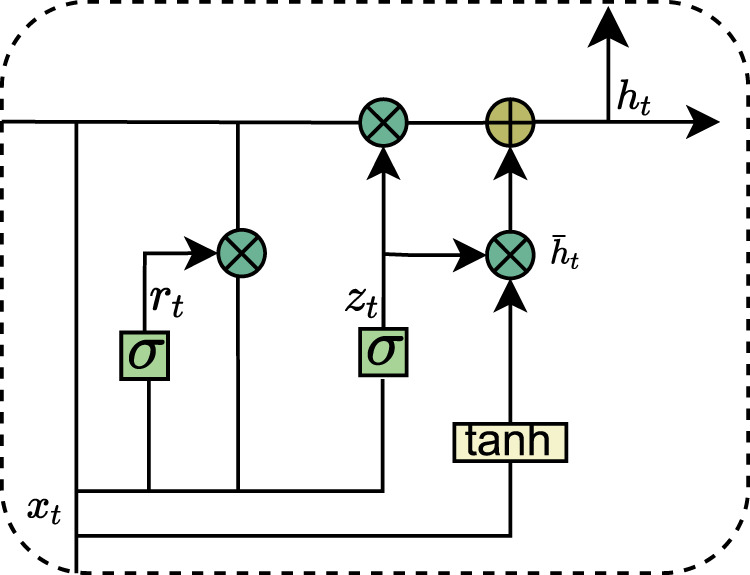
Internal structure of GRU.

Recurrent Neural Networks (RNNs) have demonstrated a strong ability to efficiently capture global information when dealing with sequential data. However, they encounter significant challenges, such as gradient vanishing and gradient explosion during the learning process, which severely hinder the model’s capacity to learn the effects of long-term dependencies in data sequences, thereby limiting performance improvement. To address these issues, researchers have proposed several advanced RNN variants aimed at more effectively learning and capturing long-term dependent features. One notable variant is the Long Short-Term Memory Network (LSTM), which selectively retains or forgets information through a gating mechanism, effectively alleviating the gradient problems and enabling the capture of long-term dependencies. However, LSTMs are associated with relatively high computational complexity. In response, the Gated Recurrent Unit (GRU) adopts a simplified gating mechanism, enhancing training efficiency while preserving the ability to manage long-term dependencies. The structure of the GRU is illustrated in Figure 4. Specifically, the GRU regulates the transfer and flow of information through two primary gating controls: the reset gate and the update gate, thereby striking a balance between efficiency and performance. The data update of the GRU is as shown in Equation 1.

$$z_{t}=\sigma \left(W_{z}x_{t}+U_{z}h_{t-1}+b_{z}\right)$$

$${\bar{h}}_{t}=\tanh\left(W_{h}x_{t}+U_{h}\left(r_{t}\cdot h_{t-1}\right)+b_{h}\right)$$ (1)

$$h_{t}=\left(1-z_{t}\right)\cdot {\bar{h}}_{t}+z_{t}\cdot {\bar{h}}_{t-1}$$

where $r_{t}$ denotes the reset gate at moment t , $z_{t}$ denotes the update gate, ${\bar{h}}_{t}$ denotes the candidate activation state, $h_{t}$ denotes the activation state, and $h_{t-1}$ is the hidden state at moment t−1 . The role of the update gate z is to decide which historical information needs to be forgotten and which new information needs to be accepted, while the reset gate r extracts information from the historical information based on the candidate states.

To enhance the learning capability, we employ a bi-directional gated recurrent unit (Bi-GRU) network. Unlike traditional gated recurrent units (GRUs), Bi-GRUs process input sequences in both directions, analyzing forward and backward sequences simultaneously to efficiently capture contextual information from both past and future states. Specifically, the Bi-GRU generates two hidden sequences: a forward sequence and a reverse sequence. These sequences are then combined to create a comprehensive representation of the overall sequence. Since the Bi-GRU can be viewed as a combination of two unidirectional GRUs, the state of the Bi-GRU at time t is derived by weighting the hidden layer state with the reverse hidden layer state: and the data update of the Bi‐GRU is as shown in Equation 2.

$$\vec{h_{t}}=GRU\left(x_{t},\vec{h_{t-1}}\right)$$

$$\overset{⃖}{h_{t}}=GRU\left(x_{t},\overset{⃖}{h_{t-1}}\right)$$ (2)

$$h_{t}=w_{t}\vec{h_{t}}+v_{t}\overset{⃖}{h_{t}}+b_{t}$$

where the function $\text{GRU}\left(\;\right)$ represents a nonlinear transformation of the input feature vector (Liang and Xiaoyong, 2019) to convert the feature vector into the hidden layer state corresponding to the GRU; $w_{t}$ and $v_{t}$ denote the forward hidden layer state weight and the forward hidden layer state weight of the BiGRU, respectively; $b_{t}$ denote the bias of the hidden layer state at the moment; $x_{t}$ are the inputs at the moment, $\vec{h_{t}}$ and $\overset{⃖}{h_{t}}$ the outputs of the hidden layer state at the moment t, and the outputs of the inverted hidden layer state, respectively.

In our research application, we employed two layers of bi-directional gated recurrent units (Bi-GRU) (Schuster and Paliwal, 1997) to model the long-term dependencies between enhancers and promoters, with each layer consisting of 50 output units. By fully leveraging the capabilities of Bi-GRUs to extract bidirectional semantic dependencies, our model demonstrates a certain improvement over unidirectional networks in terms of both accuracy and stability.

#### 2.2.3 Dynamic weighted fusion

In recent years, feature fusion has emerged as a critical component for enhancing model performance across various machine learning tasks. Existing feature fusion methods primarily rely on three approaches: 1) Fixed-weight fusion, which assigns predetermined weights to different features, failing to adapt to the varying importance of features across different samples; 2) Simple concatenation, which merely combines features without considering their relative importance, potentially leading to information redundancy; and 3) Attention fusion, which, while adaptive, often focuses solely on global feature relationships while neglecting local feature dependencies. These limitations certainly impair the model’s ability to capture complex feature interactions, especially in scenarios where feature importance varies dynamically across different DNA sequences.

**FIGURE 5:**
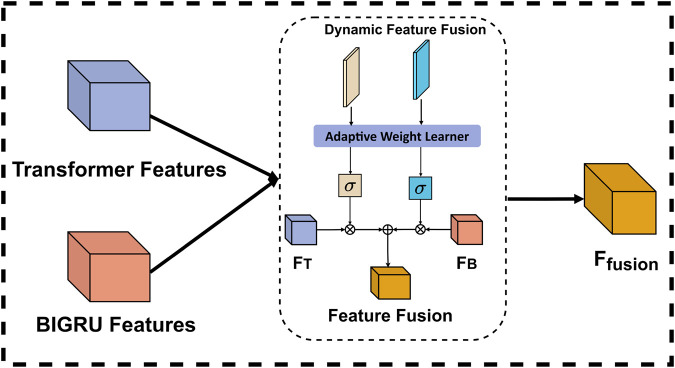
Internal structure of Dynamic feature fusion.

To overcome these limitations, we propose an adaptive weighted fusion mechanism that introduces a novel dual-stream dynamic weighting strategy. Our mechanism employs two learnable parameters, weight_transformer and weight_gru, which work in conjunction with a context-aware adaptation module. Unlike traditional fixed-weight or simple concatenation methods, our approach dynamically adjusts the significance of each feature representation based on both local sequence patterns and global contextual information. Specifically, the Transformer stream captures long-range dependencies and global contextual structures, while the BiGRU stream is better suited for modeling local and mid-range sequential patterns, due to its recurrent nature. The dynamic weighting mechanism then intelligently balances these complementary features based on their relevance to the current input sequence. The structure of dynamic feature fusion is shown in Figure 5.

In the implementation of the adaptive weight learning process, the two learnable parameters are initially set to balanced values and subsequently optimized through backpropagation during training. This approach enables the system to automatically identify the importance of each feature representation for different sequence types without human intervention. To ensure reasonable and stable weight allocation, we employ a Sigmoid function to constrain the weights within the range of [0, 1], allowing the model to flexibly adjust the influence of each feature stream based on the characteristics of the input data. During application, the model performs dynamic fusion by automatically increasing the values of weight_transformer for sequences that depend on long-distance relationships, while elevating the values of weight_gru for sequences that rely on local patterns. This weight allocation is entirely determined by the model’s internal mechanisms without explicit rule intervention, enabling our fusion mechanism to adaptively select optimal feature combinations for different types of DNA sequences, thereby effectively enhancing model performance and generalization capability. The final fusion is as shown in Equation 3.

$$h_{\text{fused}}=\sigma \left(w_{t}\right)\cdot \;h_{\text{transformer}}+\sigma \left(w_{g}\right)\cdot h_{\text{gru}}$$ (3)

Where $\sigma \left(\;\right)$ denotes the Sigmoid function, as well as $w_{g}$ and $w_{t}$ denote weight_transformer and weight_gru, respectively. $h_{\text{transformer}}$ and $h_{\text{gru}}$ then the feature representations obtained from Transformer and GRU encoding. This weighted fusion mechanism allows the model to flexibly adjust the influence of each feature representation when dealing with different types of sequences. For example, in some cases, the features generated by the Transformer may be more important, while in other cases, the context-capturing capability of the GRU may be more critical. By dynamically adjusting these weights, the model has better generalization ability and adaptability, thus improving the overall prediction performance.

#### 2.2.4 CBAM attention mechanism

The CBAM attention mechanism is a well-designed and efficient module that can be seamlessly integrated into any convolutional neural network architecture, certainly enhancing model performance while maintaining a low computational cost. This mechanism comprises two primary components: a channel attention module and a spatial attention module. Together, these modules enable CBAM to adaptively adjust the importance of each channel and spatial location within the input feature map, thereby improving the model’s capacity to characterize features and enhance decision-making accuracy. Specifically, after the original features are processed by the channel attention module, a weighted feature map is generated, which is subsequently forwarded to the spatial attention module for further processing. Ultimately, the output from the spatial attention module is another weighted feature map. This design allows the CBAM module to substantially improve the model’s ability to represent input features, thereby enhancing overall performance and effectiveness.

##### 2.2.4.1 Channel attention module

In deep learning applications, the significance of information varies considerably across different channels in the feature map. To address this issue, researchers have developed the channel attention module, which effectively processes the information within each channel of the input feature map. In conventional convolutional neural network architectures, the channel weights are relatively static and do not adapt according to the importance of the features. In contrast, the channel attention module is designed to enable the model to dynamically adjust the weights of each channel based on the relative importance of various features. This adaptive mechanism enhances the model’s ability to focus on key features more efficiently, thereby improving its overall performance.

**FIGURE 6:**
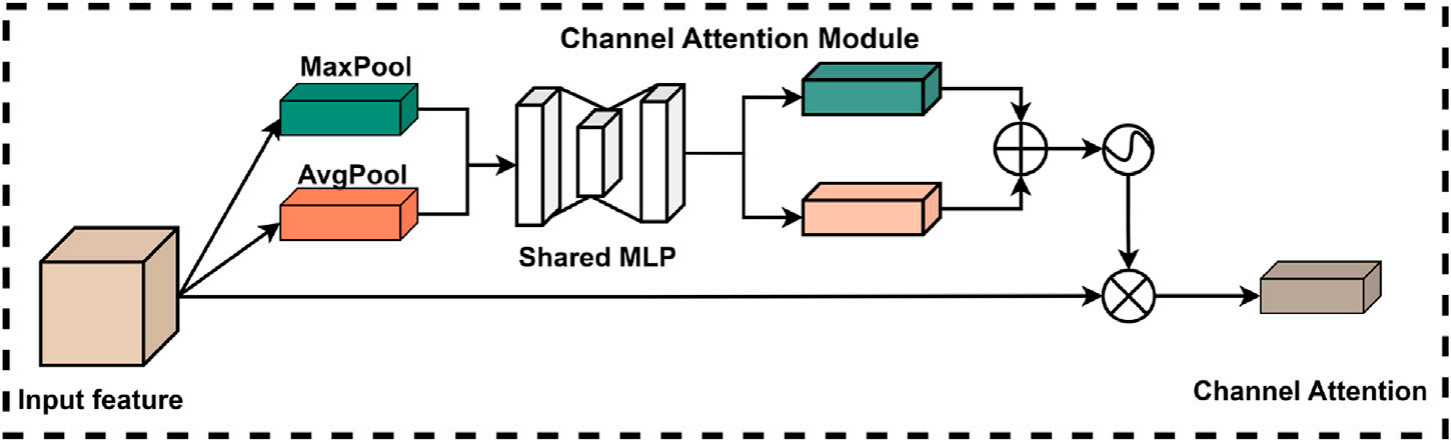
Channel attention module of CBAM.

The introduction of the channel attention module enables the model to more effectively utilize the information conveyed by each channel, resulting in certain enhancements in feature representation and overall task performance. Specifically, the structure of the channel attention module within the CBAM is illustrated in Figure 6. In this module, the original feature map is first compressed using global maximum pooling and global average pooling to produce two feature vectors with distinct channel dimensions. Subsequently, these two feature vectors are processed by a shared fully-connected neural network, which performs element-wise summation and is ultimately normalized using a sigmoid activation function. This process allows the model to derive weights for each channel. Finally, these normalized weights are multiplied by the input feature maps to effectively weight the channel features.

##### 2.2.4.2 Spatial attention module

**FIGURE 7:**
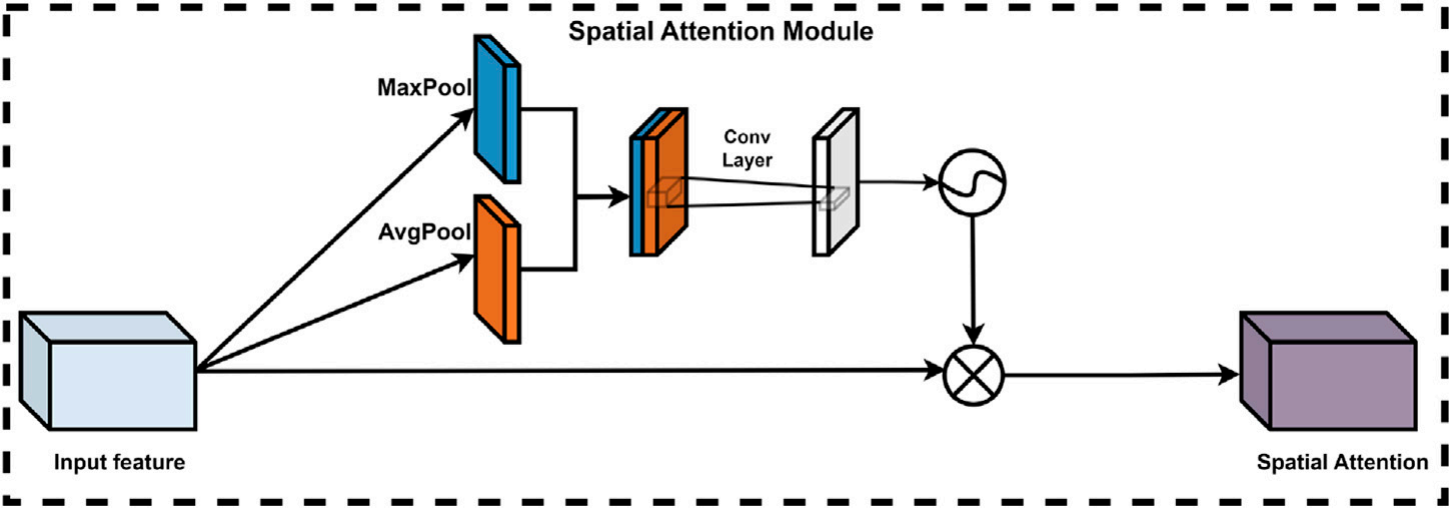
Spatial attention module of CBAM.

In deep learning models, the significance of various spatial locations within the feature map is not uniform; certain locations contribute more substantially to the model’s judgment and decision-making processes. Consequently, the integration of a spatial attention module is crucial for optimizing model performance. This module adaptively learns the weights assigned to each spatial location, thereby effectively enhancing or diminishing the importance of specific features. This process utilizes the output feature map from the channel attention module. Specifically, the spatial attention module processes the input feature maps by first compressing the features along the channel dimension through global maximum pooling and global average pooling operations, resulting in two representative feature matrices. These matrices are then concatenated and processed by a convolutional neural network to produce a training feature matrix, followed by the application of a sigmoid activation function to derive the corresponding spatial attention weights. Ultimately, the final feature map is obtained by performing an element-wise multiplication of the spatial attention weights with the input feature matrix. The spatial attention module (Lin et al., 2024) is depicted in Figure 7.

In summary, the spatial attention module certainly enhancing model performance while maintaining a low computational cost. Influences the importance assigned to each position within the feature map. This enhancement not only improves the representation of the feature map but also markedly boosts the overall performance of the model.

#### 2.2.5 Sequence prediction

In this study, we designed a model that incorporates a fully connected layer with 100 neurons, aimed at extracting and generalizing input features while mapping these features to the predicted output space. This design enables improved identification of potential patterns within the data, thereby providing a robust foundation for subsequent analyses. To effectively mitigate the risk of overfitting, we implemented the Batch Normalization method to standardize inputs, which enhances both the stability and speed of the training process. Concurrently, we employed the Dropout technique, which randomly discards a certain percentage of neurons. This approach not only reduces the model’s reliance on the training data but also bolsters its generalization capabilities. The choice of activation function is also a critical aspect of the model’s design. Following the Batch Normalization process, we utilized the ReLu activation function, a nonlinear function adept at capturing complex relationships within the input data, thereby improving the model’s feature learning capacity. Ultimately, the feature vector extracted through the Fully Connected Layer is directed to a single neuron output layer equipped with a sigmoid activation function. This design facilitates the model’s ability to output a probability value that reflects the likelihood of interactions between samples.

## 3 Model training and testing

The interaction between enhancers and promoters is influenced by the specific characteristics of each cell line, highlighting the substantial differences that exist among them. Consequently, models developed from training on one cell line may not yield satisfactory performance on others. To address this issue, we trained and evaluated models for each cell line individually, thereby ensuring the accurate capture of their respective unique interaction patterns.

To maintain comparability with existing models (e.g., EPI-DLMH, SPEID, PEP-WORD, EPIVAN, SIMCNN, EPI-Mind, and EPI-Trans), we used the same dataset partitioning and training process as in previous studies. Given the cell line specificity of EPI, we developed six independent models, ensuring that each model was trained on a different cell line. By using the same training and test sets for each cell line as in previous studies, we were able to ensure a fair performance comparison with existing methods. Next, we detail the specific training methods used for each cell line:

(1) For each cell line, the initial unbalanced dataset was named $D_{\text{imb}}$ . Through stratified sampling $D_{\text{imb}}$ was divided into a training set and a test set, where 90% of the data were used for the training set $D_{\text{itr}}$ and 10% for the test set $D_{\text{test}}$ to ensure that the category distributions remained consistent across the training and test data.
(2) To cope with the category imbalance problem, a new balanced training set $D_{\text{train}}$ is constructed by oversampling a few class samples in the training set $D_{\text{itr}}$ by a factor of 20.
(3) Before training the model, 5% of the data in the $D_{\text{train}}$ is used as a validation set $D_{\text{val}}$ for optimizing the hyperparameters of the model and selecting an appropriate optimization algorithm.
(4) Models were trained on the training set $D_{\text{train}}$ for each cell line and validated on the validation set $D_{\text{val}}$ to ensure that the models performed optimally.
(5) After validation, the final model is tested on the test set $D_{\text{test}}$ .
(6) The performance of the model was thoroughly evaluated using the area under the subject operating characteristic curve (AUROC) and the area under the precision-recall curve (AUPR) as evaluation metrics.

### 3.1 System implementation and configurations

As an open-source automated hyperparameter optimization framework, Optuna (Akiba et al., 2019) provides users with powerful model tuning tools. The framework adopts a command-based, run-defined API design that supports the dynamic construction of hyperparameter search spaces, enabling researchers to flexibly define and tune optimization goals. Optuna integrates a variety of advanced optimization algorithms, including TPE and CMA-ES, and seamlessly works with mainstream machine learning frameworks such as TensorFlow and PyTorch, significantly enhancing its applicability in the field of deep learning. By defining clear optimization objectives and search spaces, Optuna can automatically execute the hyperparameter optimization process and systematically explore parameter combinations to help users discover the optimal model configuration. This automated optimization mechanism not only saves considerable time in manual parameter tuning but also effectively improves the overall performance of the machine learning model, enabling it to exhibit stronger predictive ability and generalization performance across various application scenarios.

In our implementation, we use Optuna to optimize the learning rate (l), training period (e), and training batch size (b). We set the optimization range for l to include 5e-5, 1e-5, 5e-4, 1e-4, 5e-3, 1e-3, 5e-2, 1e-2, and 1e-1. The optimization range for e is from 20 to 100, with a step size of 10, while the optimization range for b is from 16 to 64. The final optimal parameters for the learning rate (l), training period (e), and training batch size (b) are 1e-3, 30, and 64, respectively.

Based on these optimal metrics, we conducted extensive experiments with various optimizers, including NAdam, Adam, and RMSprop. The model was trained on a server equipped with a GeForce RTX 4080 GPU, featuring 16 GB of RAM and a total of 128 GB of memory. The server operates on Windows, with installed software including CUDA 11.8, Conda 4.7.10, and Python 3.8. After evaluating the average AUPR and AUROC, we ultimately selected NAdam as the final optimizer and established an early stopping criterion, which halts training if there is no decrease in loss after five epochs. The specific metrics of the optimizer are detailed in Tables 8 and 9 of Supplementary Material S1.

## 4 Evaluation metrics

When evaluating classification models on unbalanced datasets, it is crucial to select appropriate metrics. In this context, AUROC (Area Under the Receiver Operating Characteristic Curve) (Hanley and McNeil, 1982) and AUPR (Area Under the Precision-Recall Curve) (Davis and Goadrich, 2006) are particularly significant as evaluation metrics. A distinctive feature of AUROC is its ability to assess model performance independently of the sample distribution imbalance. Typically, an AUROC value close to 1 indicates that the model performs exceptionally well, effectively distinguishing between positive and negative samples. Conversely, AUPR offers an alternative perspective by illustrating the trade-off between the model’s accuracy in identifying positive examples and its coverage capability. Thus, an AUPR value near 1 similarly signifies superior model performance. The combination of these two metrics facilitates a comprehensive assessment of model performance from different angles, thereby enhancing the reliability and comparability of experimental results. This holistic evaluation approach not only aids researchers in understanding model performance on unbalanced datasets but also provides a robust foundation for future related studies. Consequently, the selection of AUROC and AUPR as assessment indicators not only accurately reflects model performance on unbalanced datasets but also ensures the persuasiveness and comparability of the experimental findings.

## 5 Results and discussion

### 5.1 Comparison of different modeling frameworks

In this study, we conducted ablation experiments on the network architecture of the proposed model to evaluate the impact of various modules. Specifically, we compared the performance of several network combinations, including CNN, CNN + Transformer, and CNN models enhanced with advanced components such as BiGRU, CBAM, and DynFusion. Additionally, we established the underlying CNN structure as a baseline for benchmarking the models. The experimental results, presented in Tables 2 and 3, indicate a certain improvement in the performance of the combination of CNN, Transformer, BiGRU, DynFusion, and CBAM modules compared to the baseline CNN model and the version with fewer integrated components.

**TABLE 2:** AUPR results for different architectural approaches.

Model/Cell lines	GM12878	HUVEC	HeLa-S3	IMR90	K562	NHEK
CNN	0.591	0.641	0.722	0.660	0.636	0.885
CNN + Transformer	0.792	0.749	0.862	**0.744**	0.775	0.935
CNN + CBAM	0.743	0.603	0.766	0.681	0.717	0.837
CNN + BiGRU	0.775	0.717	0.841	0.719	0.772	**0.939**
CNN + Transformer + BiGRU	0.782	0.716	0.852	0.694	0.751	0.927
CNN + Transformer + BiGRU + CBAM	0.769	0.694	0.848	0.716	0.772	0.921
CNN + Transformer + BiGRU + DynFusion	0.799	0.743	0.863	0.730	0.782	0.923
CNN + Transformer + BiGRU + DynFusion + CBAM	**0.803**	**0.744**	**0.868**	0.741	**0.788**	0.930

**TABLE 3:** AUROC results for different architectural approaches.

Model/Cell lines	GM12878	HUVEC	HeLa-S3	IMR90	K562	NHEK
CNN	0.916	0.931	0.960	**0.919**	0.927	0.986
CNN + Transformer	0.936	0.932	0.962	0.924	0.938	**0.990**
CNN + CBAM	0.916	0.915	0.941	0.881	0.937	0.982
CNN + BiGRU	0.907	0.913	0.935	0.871	0.933	0.989
CNN + Transformer + BiGRU	0.909	0.915	0.940	0.886	0.928	0.985
CNN + Transformer + BiGRU + CBAM	0.919	0.915	0.946	0.892	0.928	0.987
CNN + Transformer + BiGRU + DynFusion	0.925	0.923	0.971	0.912	**0.944**	0.987
CNN + Transformer + BiGRU + DynFusion + CBAM	**0.932**	**0.947**	**0.972**	0.910	0.940	0.987

The selection of these specific modules was based on their unique advantages in genomic sequence analysis. We chose Transformer for its superior capability in handling long-sequence dependencies, which is crucial in genomic sequence analysis (Vaswani et al., 2017). BiGRU was selected for its ability to capture bidirectional sequence information flow, simultaneously considering both forward and backward dependencies (Jun-Young et al., 2014). The DynFusion module was designed to overcome the limitations of traditional feature fusion methods (such as simple concatenation or weighted averaging) by providing more flexible feature integration strategies (Misra et al., 2016). CBAM was chosen over other attention mechanisms due to its ability to perform feature recalibration in both channel and spatial dimensions, better capturing position-related patterns in genomic sequences (Woo et al., 2018).

Experimental results validate our architectural choices and indicate that the integration of the Transformer architecture resulted in significant improvements over the baseline CNN model across multiple cell lines, particularly in GM12878, where the area under the AUPR increased by 34.0%. The inclusion of the BiGRU further enhanced performance, yielding a 31.1% improvement in GM12878 and achieving the highest AUPR of 0.939, which represents a 6.1% increase in the NHEK cell line.

Notably, our complete model (CNN + Transformer + BiGRU + DynFusion + CBAM) demonstrated optimal performance across most cell lines, with a 35.9% enhancement in AUPR and a 1.7% increase in the AUROC in GM12878, as well as a 20.2% improvement in AUPR and a 1.3% increase in AUROC in HeLa-S3. These performance variations likely stem from cell type-specific biological characteristics. For instance, GM12878, as a lymphoblastoid cells, exhibits distinctive chromatin accessibility patterns and extensive long-range enhancer-promoter interactions, potentially making the Transformer architecture particularly effective in capturing its long-range dependencies (Jason and Kellis, 2012; Jesse et al., 2012). In contrast, HUVEC endothelial cells display relatively conservative regulatory patterns, explaining why the introduction of attention mechanisms (CBAM) yielded a 16.1% improvement in AUROC, though the overall enhancement was less pronounced than in GM12878 (Nathan et al., 2013).

The integration of DynFusion and CBAM modules has further enhanced model performance, with variations noted among different cell lines. We hypothesize that these discrepancies are linked to cell type-specific transcription factor binding patterns and distributions of epigenetic modifications. For instance, the certain performance improvement observed in GM12878 (a 35.9% increase in AUPR) may reflect the complex transcriptional regulatory networks present in this cell line, where the DynFusion module effectively integrates these multi-level regulatory signals (Žiga et al., 2021). These comprehensive enhancements across various metrics and cell lines not only validate the effectiveness of our proposed architectural design but also illuminate potential connections between model components and the underlying biological mechanisms.

### 5.2 Comparison of different feature fusion methods

Feature fusion techniques have emerged as a crucial component in deep learning, enabling the integration of information from different sources or levels to create more comprehensive data representations. In the field of bioinformatics, these fusion strategies have been widely adopted for various predictive tasks. In this study, we systematically evaluate four feature fusion strategies integrated with the CBAM within the CNN + Transformer + BiGRU architecture:

(1) Fixed Weight Fusion: This approach combines the features of the Transformer and BiGRU by means of predetermined fixed weights. For instance, in drug-target interaction prediction, DeepDTI (Tian et al., 2020) employs this method to merge drug and protein features. The weights remain constant during both training and inference, making it computationally efficient but potentially less adaptive to varying input patterns.
(2) Simple Concatenation: This straightforward method allows the feature vectors of the Transformer and BiGRU to be concatenated into a longer vector directly. DeepSEA (Jian and Olga, 2015) successfully utilized this approach to combine DNA sequence features extracted from different convolutional layers. While simple to implement, this method treats all features equally without considering their relative importance.
(3) Attention Fusion: This strategy utilises the attention mechanism to dynamically assign importance weights to the features of Transformer and BiGRU. For example, iEnhancer-ECNN (Nguyen et al., 2019) leverages attention mechanisms in enhancer recognition tasks to fuse multi-scale features. This approach allows the model to focus on more relevant features depending on the input context.
(4) Dynamic Fusion: This advanced approach adaptively adjusts fusion strategies based on input characteristics. Through the implementation of similar dynamic feature fusion methods, the MIDF-DMAP (Yi et al., 2025) model has demonstrated its effectiveness in predicting drug molecular activity. While this approach offers maximum flexibility, it requires careful design to maintain computational efficiency.

**FIGURE 8:**
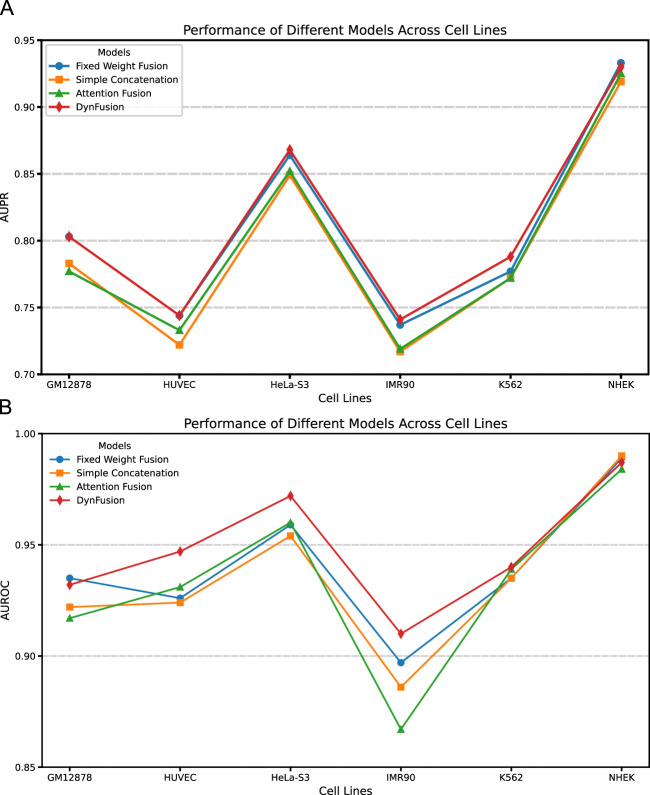
AUPR and AUROC results of different feature fusion methods on six cell lines. **(A)** AUPR scores of different fusion methods across datasets. **(B)** AUROC scores of different fusion methods across datasets. Among the evaluated methods, DynFusion (red) consistently demonstrates superior performance across most cell lines. It achieves the highest AUPR on K562 and NHEK, and an AUROC value approaching 1 on both HUVEC and K562. In contrast, the Fixed Weight Fusion method (blue) exhibits suboptimal performance in terms of AUPR, particularly on GM12878 and IMR90. Simple Concatenation (green) and Attention Fusion (orange) show comparable results across most cell lines but slightly lag behind DynFusion. Overall, DynFusion outperforms all other fusion strategies across all datasets, with a particularly pronounced advantage observed in the NHEK cell line.

Through an in-depth analysis of the area under the precision-recall curve (AUPR) and the area under the receiver operating characteristic curve (AUROC) for six cell lines (GM12878, HUVEC, HeLa-S3, IMR90, K562, and NHEK), as illustrated in Figures 8A,B, we observe performance improvements from dynamic feature fusion. These improvements align closely with the dynamic nature of enhancer-promoter interactions. Specifically, regarding AUPR, the dynamic fusion method exhibited enhancements over simple concatenation: in the GM12878 cell line, performance increased by approximately 2.6%; in HUVEC, by 2.2%; and in HeLa-S3, by 1.9%. These differential improvements likely reflect the distinct regulatory patterns of enhancer-promoter interactions across cell types. For instance, the higher performance gain in GM12878 may be attributed to its more complex chromatin loop structures and frequent long-range regulatory events (Jesse et al., 2012; Schoenfelder and Fraser, 2019), where dynamic fusion demonstrates superior capability in integrating both long-range and local features.

Similarly, for the AUROC metric, the dynamic fusion method demonstrated superior performance, with improvements of approximately 2.5% in HUVEC and 1.2% in HeLa-S3. These improvements can be attributed to dynamic fusion’s ability to adaptively adjust feature weights, thereby better capturing cell-specific chromatin conformational changes and transcription factor binding patterns (Nathan et al., 2013; Anshul et al., 2015). Notably, in the K562 cell line, the dynamic fusion method improved AUPR by approximately 1.6% and AUROC by 0.1% compared to attention fusion. As a leukemia cell line, K562 exhibits unique characteristics in its gene regulatory networks, and the relatively modest performance improvements may reflect its more stable chromatin structure and regulatory mechanisms (Dunham et al., 2012). The consistent performance improvements across different cell lines not only underscore the strong cross-cell line applicability of the dynamic fusion approach but also indicate its effectiveness in adapting to the biological characteristics of enhancer-promoter interactions across different cell types.

### 5.3 Impact of data imbalance on modeling

Data imbalance poses a significant challenge in EPIs prediction. In our dataset, the ratio of positive to negative samples is approximately 1:20, leading to a pronounced tendency for the model to predict negative samples during training. This imbalance adversely affects the model’s ability to recognize rare positive samples. Experimental results indicate that the average area AUPR of the model is notably lower than that of the AUROC, with values of 0.644 and 0.920, respectively, when using the original dataset without oversampling. This discrepancy highlights the model’s limited capacity to identify positive samples in the context of unbalanced data.

To address this issue, we systematically evaluated six oversampling strategies: 0×, 5×, 10×, 15×, 20×, and 25×, and assessed their performance across six cell lines (GM12878, HUVEC, HeLa-S3, IMR90, K562, NHEK). The results shown in Tables 10 and 11 of Supplementary Material S1 indicate that, in the vast majority of cell lines, 20-fold oversampling achieved the best overall performance in terms of AUROC and AUPR metrics. For instance, in the GM12878 cell line, AUROC improved from 0.919 to 0.932, while AUPR significantly increased from 0.708 to 0.803, representing a 13.4% enhancement. Similarly, in cell lines such as HUVEC, IMR90, K562, and NHEK, 20-fold oversampling resulted in substantial performance improvements, particularly in AUPR, indicating enhanced recognition of positive samples.

However, oversampling may also lead to saturation or even a slight decrease in performance. In the GM12878, HUVEC, HeLa-S3, K562, and NHEK cell lines, a 25-fold oversampling did not further enhance the AUPR; in some instances, it even resulted in a slight decrease (e.g., from 0.744 to 0.699 for HUVEC, and from 0.788 to 0.718 for K562). This suggests that excessive replication of positive samples may induce overfitting in the model, thereby impairing its generalization performance. In summary, the 20-fold oversampling strategy effectively avoids overfitting while maintaining performance improvements, more efficiently mitigating the adverse effects of category imbalance, and significantly enhancing the predictive balance of the model with respect to positive and negative samples. Although a 20-fold increase was optimal for most cell lines, variations in the optimal oversampling factor were noted among different cell lines, which may be attributed to the complexities of their gene regulatory networks and the inherent distributional characteristics of the positive samples. Future research will further investigate cell line-specific adaptive oversampling strategies for more refined model optimization and will also consider integrating other data enhancement methods (e.g., SMOTE, ADASYN, *etc.*) to bolster the robustness and generalization capabilities of the model under conditions of data imbalance.

### 5.4 Performance analysis of cell specific models

Enhancer-promoter interactions exhibit distinct properties across various cell lines, suggesting certain differences in interaction patterns. Consequently, models developed based on a specific cell line for training are often unsuitable for direct application to other cell lines. In this study, we developed a model specifically trained for a particular cell line, referred to as EPI-DynFusion-spe. Tables 4 and 5 present the AUPR and AUROC results for this specific model. The findings indicate that the EPI-DynFusion-spe model performs effectively in predicting enhancer-promoter interactions (EPI) when both the training and test sets originate from the same cell line. For instance, when the models were trained and tested on the same cell lines, the GM12878, HUVEC, HeLa-S3, IMR90, K562, and NHEK cell lines achieved AUROC values of 0.932, 0.947, 0.972, 0.910, 0.940, and 0.987, respectively, while the AUPR values were 0.803, 0.744, 0.868, 0.741, 0.788, and 0.930. These results demonstrate that the model exhibits high prediction accuracy when both the training and test sets are derived from the same cell line.

**TABLE 4:** AUPR results of EPI-DynFusion-spe across different cell lines.

Train/Test cell lines	GM12878	HUVEC	HeLa-S3	IMR90	K562	NHEK
GM12878	**0.803**	0.091	0.088	0.080	0.134	0.094
HUVEC	0.154	**0.744**	0.212	0.097	0.152	0.146
HeLa-S3	0.111	0.204	**0.868**	0.082	0.209	0.143
IMR90	0.179	0.159	0.097	**0.741**	0.138	0.130
K562	0.106	0.135	0.093	0.072	**0.788**	0.123
NHEK	0.102	0.182	0.165	0.111	0.138	**0.930**

**TABLE 5:** AUROC results of EPI-DynFusion-spe across different cell lines.

Train/test cell lines	GM12878	HUVEC	HeLa-S3	IMR90	K562	NHEK
GM12878	**0.932**	0.613	0.541	0.542	0.596	0.568
HUVEC	0.618	**0.947**	0.634	0.556	0.589	0.598
HeLa-S3	0.596	0.637	**0.972**	0.527	0.662	0.591
IMR90	0.612	0.612	0.573	**0.910**	0.617	0.517
K562	0.564	0.620	0.557	0.518	**0.940**	0.553
NHEK	0.595	0.629	0.610	0.569	0.615	**0.987**

However, when evaluated across different cell lines, the model’s performance shows a noticeable decline compared to scenarios where training and testing are conducted within the same cell line. In contrast, other existing models, such as SPEID, EPI-DLMH, EPI-Mind, and EPI-Trans, exhibited similar trends. This observation further indicates that relying solely on sequence information from a specific cell line is ineffective for predicting the role of promoters at enhancers in other cell lines. These models are limited to learning the interaction patterns between enhancers and promoters within a given cell line. Consequently, we can conclude that enhancer-promoter interactions are distinctly cell line-specific, which fundamentally limits the ability to make predictions across different cell lines.

### 5.5 Computational complexity of the EPI-DynFusion model

In this section, we provide insights into the computational aspects of each module of the proposed EPI-DynFusion model and compare it with baseline models. The training and testing times for each cell line are detailed in Supplementary Material S1: Tables 12–15. Table 12 presents the average training and testing times, offering an overview of all samples across the six cell lines. A comparative analysis of the average training duration for all samples indicates that the EPI-DynFusion-gen model requires a longer training duration. This extended duration is attributed to the incorporation of a large number of samples from all cell lines during training. Table 13 reveals that the average training time for the EPI-DynFusion model is 33.2 min, which is shorter than that of the baseline model EPI-Trans at 35.0 min, thereby demonstrating higher training efficiency. Furthermore, in comparison to other baseline models such as EPI-Mind and EPI-DLMH, EPI-DynFusion exhibits an advantage in training time; although these models have shorter training durations, their accuracy and performance may be constrained. Consequently, EPI-DynFusion achieves a better balance between training time and performance.

Regarding memory usage, Tables 14 and 15 illustrate the memory consumption of different models across the six cell lines. EPI-DynFusion’s memory usage stands at 4,995.72 MB, slightly higher than that of EPI-Trans at 3,652.52 MB, yet lower than the CNN + Transformer combination, which has a memory requirement of 5,012.50 MB due to the inclusion of the Transformer module. Despite the increased memory consumption associated with the Transformer module, the performance and training time advantages of the EPI-DynFusion model ensure that its memory requirements remain within an acceptable range. Overall, EPI-DynFusion offers a superior trade-off between training efficiency and memory consumption.

### 5.6 LOCO cross-validation

To rigorously evaluate the generalization ability of EPI-DynFusion and mitigate the risk of sequence homology-based data leakage, this study employs the chromosome Leave-One-Cross-Validation (LOCO) strategy proposed by Tahir et al. (2025). The LOCO method segments the dataset based on chromosome location, utilizing all chromosome data except for the target chromosome to train the model, while testing it on the retained chromosome. This approach effectively avoids the sequence homology bias that may arise from traditional random segmentation methods, thereby providing a more realistic and reliable performance evaluation framework for sequence-based EPI prediction. In this experiment, LOCO validation was conducted on all 23 human chromosomes (autosomes 1–22 and the X chromosome), and the average AUROC values (expressed as mean ± standard deviation) were calculated for each cell line. Table 6 presents the results of the performance comparison of EPI-DynFusion with five baseline methods across six human cell lines (see Supplementary Material S1: Tables 2-7 for detailed results for each chromosome).

**TABLE 6:** AUROC metrics for six models under LOCO split cross-validation.

Model/Cell lines	GM12878	HeLa-S3	HUVEC	IMR90	K562	NHEK
EPI-VAN	0.587 ± 0.043	0.624 ± 0.091	0.596 ± 0.072	0.586 ± 0.078	0.560 ± 0.058	0.561 ± 0.088
EPI-DLMH	0.582 ± 0.042	0.637 ± 0.086	0.607 ± 0.060	0.585 ± 0.061	0.595 ± 0.064	0.587 ± 0.088
EPI-Mind	0.582 ± 0.056	0.598 ± 0.057	0.602 ± 0.062	0.607 ± 0.111	0.580 ± 0.074	0.596 ± 0.058
EPI-Trans	0.608 ± 0.052	0.655 ± 0.060	0.632 ± 0.082	0.629 ± 0.091	0.607 ± 0.078	0.610 ± 0.062
LOCO-EPI	0.565 ± 0.068	0.608 ± 0.120	0.591 ± 0.105	0.593 ± 0.119	0.569 ± 0.106	0.576 ± 0.081
EPI-DynFusion	**0.614 ± 0.046**	**0.657 ± 0.057**	**0.643 ± 0.056**	**0.668 ± 0.086**	**0.641 ± 0.076**	**0.647 ± 0.052**

**TABLE 7:** EPI-DynFusion-gen model results for AUROC and AUPR.

Cell lines	GM12878	HUVEC	HeLa-S3	IMR90	K562	NHEK
AUPR	0.740	0.620	0.767	0.652	0.708	0.782
AUROC	0.944	0.939	0.958	0.931	0.948	0.980

The experimental results indicate that EPI-DynFusion achieved optimal performance across all tested cell lines, with AUROC values ranging from 0.614 to 0.668. Compared to the LOCO-EPI baseline method, EPI-DynFusion demonstrated performance improvements of 8.7%, 8.1%, 8.8%, 12.6%, 12.7%, and 12.3% in the IMR90, K562, and NHEK cell lines, respectively. Under the LOCO evaluation framework, the performance of all models decreased compared to random data partitioning, confirming the inherent challenges of cross-chromosome generalization tasks. However, EPI-DynFusion exhibited relatively superior robustness, achieving higher average AUROC values while demonstrating smaller performance variations across different chromosomes. Specifically, in the IMR90 (0.668 ± 0.086) and K562 (0.641 ± 0.076) cell lines, EPI-DynFusion’s performance surpassed that of the next-best method, EPI-Trans (0.629 ± 0.091 and 0.607 ± 0.078, respectively).

These results indicate that the dynamic fusion mechanism employed by EPI-DynFusion effectively integrates the complementary feature representations of multiple neural network modules, thereby enhancing the model’s ability to capture cross-chromosomal biological signals, rather than relying solely on local sequence homology information. This exceptional generalization performance provides robust support for the efficacy of EPI-DynFusion in practical genomics applications.

### 5.7 Performance of a generic model trained on all cell lines

In a previous study, we trained a specific model for all cell lines, referred to as EPI-DynFusion-spe. While this model, developed individually for each cell line, effectively captured unique features, it demonstrated suboptimal performance when identifying characteristics across different cell lines. Therefore, we propose a new general model designed to improve overall efficiency by integrating features shared among various cell lines. In constructing this generic model, we based our work on several assumptions: First, through deep learning methodologies, our model aims to extract key features relevant to EPI prediction from the available data, enabling the identification of crucial regulatory elements. Second, considering the inherent diversity of cellular phenotypes, we anticipate that certain features may exhibit variability across different cell lines, reflecting the unique biological characteristics of each cell type. Third, based on fundamental biological principles, we postulate the existence of common regulatory patterns that are preserved across different cell lines, representing potentially conserved mechanisms of gene regulation. Building on these assumptions, we have developed a new generic model named EPI-DynFusion-gen. Its training steps are as follows:

(1) The training data of the six cell lines were disrupted and mixed, and then integrated to form a new training set named $D_{\text{train}\_\text{all}}$ .
(2) Prior to training, 5% of the data from $D_{\text{train}\_\text{all}}$ was randomly selected as the validation set $D_{\text{Val}\_\text{all}}$ .
(3) Train the model on $D_{\text{train}\_\text{all}}$ and evaluate it on the $D_{\text{test}}$ test set.
(4) Still using AUROC and AUPR as performance indicators.

The AUPR results for this model across six cell lines were 0.740, 0.620, 0.767, 0.652, 0.708, and 0.782, corresponding to GM12878, HUVEC, HeLa-S3, IMR90, K562, and NHEK, respectively. The corresponding AUROC results were 0.944, 0.939, 0.958, 0.931, 0.948, and 0.980. Please refer to Table 7 for further details. By comparing the AUROC of the EPI-DynFusion-gen model with that of the EPI-DynFusion-spe model (as shown in Table 8), we found that the generic model outperformed the specific model in three cell lines: GM12878, IMR90, and K562. This observation can be attributed to the greater number of shared features present in these cell lines, which allows the generic model to extract information more efficiently. Conversely, in the other cell lines, such as NHEK and HUVEC, specific features are more pronounced. Consequently, the individually trained specific models are better equipped to capture these features, resulting in superior performance.

**TABLE 8:** Comparison of AUPR metrics for the three models.

Model/Cell lines	GM12878	HUVEC	HeLa-S3	IMR90	K562	NHEK
EPI-DynFusion-spe	0.803	0.744	0.868	0.741	0.788	**0.930**
EPI-DynFusion-gen	0.740	0.620	0.767	0.652	0.708	0.781
EPI-DynFusion-best	**0.821**	**0.782**	**0.872**	**0.794**	**0.804**	0.924

**FIGURE 9:**
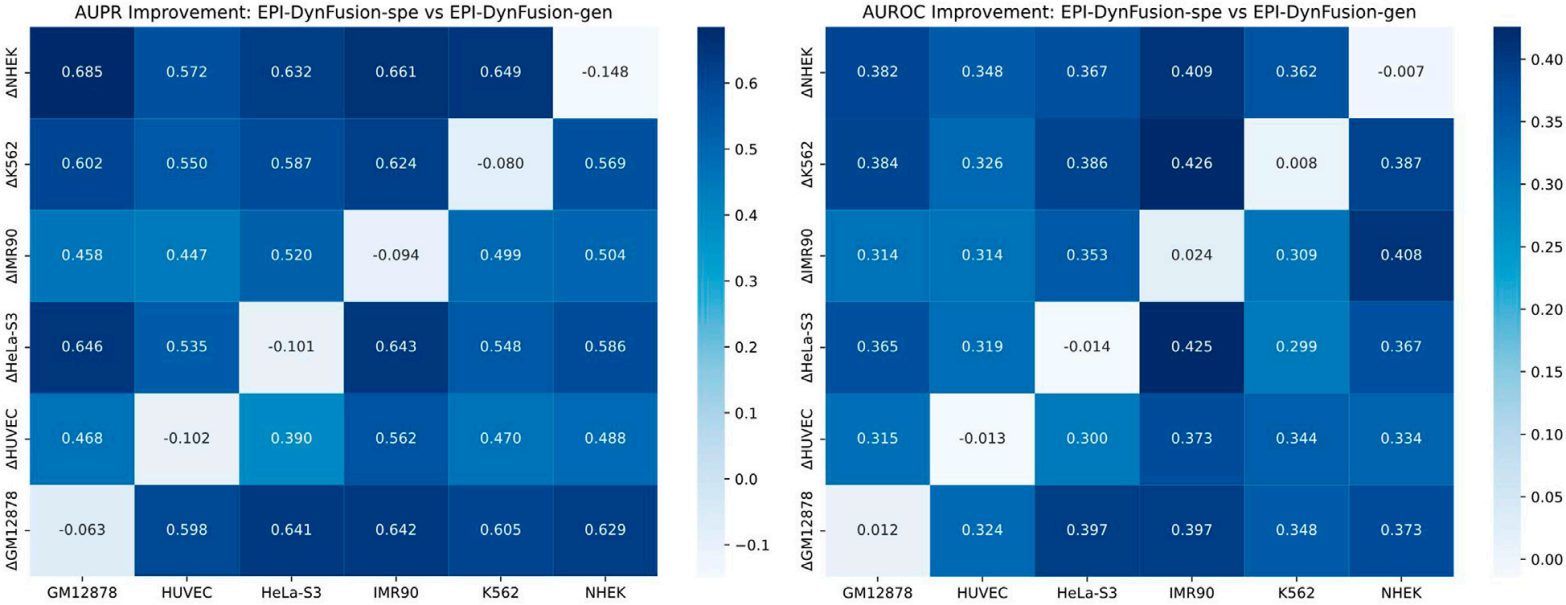
Comparison of the performance of EPI-DynFusion-spe and EPI-DynFusion-gen in cross-cell line prediction. Heatmaps of AUPR improvement (left) and AUROC improvement (right) illustrate the performance gain of EPI-DynFusion-gen compared to EPI-DynFusion-spe. The values in the heatmaps represent the performance difference (ΔPerformance = Performance_gen ‐ Performance_spe). The analysis includes six human cell lines, where the vertical axis represents the training cell line and the horizontal axis represents the test cell line. Color intensity indicates the level of improvement, with dark blue representing greater performance gains (see color scale). In cross-cell lineage evaluation, the EPI‐DynFusion-gen model showed more consistent performance improvement in both AUPR and AUROC metrics compared to the EPI‐DynFusion‐spe model in cross‐cell lineage testing.

**FIGURE 10:**
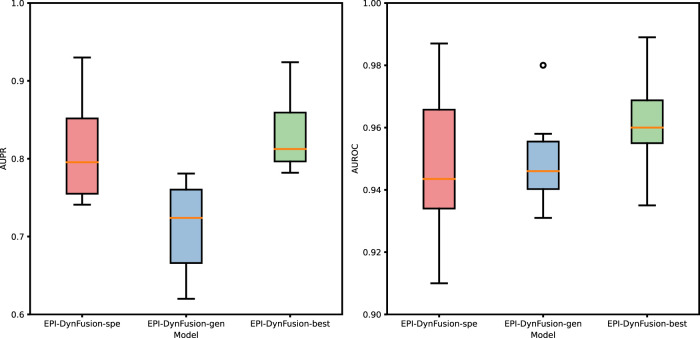
Performance evaluation of three different models through the test set. The graphs illustrate a comparison of the performance of three models based on the AUPR and AUROC metrics. The analysis includes six human cell lines. The best results were achieved by EPI‐DynFusion‐best, in contrast to the subpar outcomes observed for EPI‐DynFusion‐gen.

To evaluate the cross-cell line predictive ability of our proposed method, we analyzed the performance differences between the original EPI-DynFusion-spe model and the EPI-DynFusion-gen model. Figure 9 presents a heatmap illustrating the differences in AUROC and AUPR between the two models across various cell lines. The x-axis denotes the cell lines, while the y-axis indicates the metric differences between the fine-tuned EPI-DynFusion-gen model, which is customized for the target cell line, and the original EPI-DynFusion-spe model. The heatmaps depicted in Figure 10 reveal that the majority of blocks are dark-colored, signifying a substantial enhancement in cross-cell lineage prediction performance achieved by the EPI-DynFusion-gen model compared to the original EPI-DynFusion-spe model. Although the heatmap indicates that the EPI-DynFusion-gen model generally enhances cross-cell line prediction performance, there are instances where the gen model performs worse than the spe model. This observation aligns with our initial assumptions regarding the inherent diversity of cellular phenotypes. This phenomenon can be explained from two perspectives: model characteristics and cell line specificity. The EPI-DynFusion-gen model excels at capturing common features and patterns across different cell lines, supporting our hypothesis about the existence of conserved regulatory patterns. In contrast, the EPI-DynFusion-spe model focuses on identifying and learning unique features specific to individual cell lines, consistent with our understanding of cell-type-specific characteristics. Furthermore, EPI binding features may exhibit variability across different cell lines, where certain sites serve as EPI binding sites in one cell line but not in others. This variability validates our initial assumption about feature differences across cell lines. This cell line-specific variation, combined with the potential trade-off in the gen model’s prediction accuracy for specific cell lines in favor of enhanced generalization capability, collectively explains the observed performance patterns in cross-cell line predictions. It also demonstrates the complex balance between capturing shared regulatory mechanisms and preserving cell-type-specific features.

Although EPI-DynFusion-gen did not perform optimally across all cell lines, it demonstrated the potential to capture common features shared among multiple cell lines. Based on this observation, the next phase of the study will involve designing a model capable of both extracting shared features and identifying cell line-specific characteristics, thereby enhancing the accuracy of EPI predictions and accommodating the diverse features present in different cell lines.

### 5.8 Optimal models for predicting EPIs

To enhance the performance of the EPI-DynFusion model, we have drawn important insights from the EPI-DynFusion-gen model and propose an improved version, the EPI-DynFusion-best model. While the EPI-DynFusion-gen effectively captures generic features of cell lines through pre-training on a comprehensive dataset of enhancer-promoter interactions, it struggles to identify cell line-specific characteristics. Based on this observation, we hypothesized that by making the model learn specific features more efficiently through a two-stage training approach, its performance might be improved.

In our two-stage training strategy, the first stage focuses on learning generic enhancer-promoter interaction patterns shared across different cell lines, establishing a robust foundation for feature extraction. The second stage then fine-tunes the model on cell line-specific data, allowing it to adapt its feature detectors to capture unique regulatory patterns. Future work could further validate this approach through various interpretability techniques, such as attention visualization to track the model’s focus shifts during fine-tuning, feature importance analysis to identify cell type-specific genomic signatures, and comparison of activation patterns between generic and specialized models to understand the evolution of feature recognition. We anticipate that such analyses could reveal how pre-training helps the model develop sensitive detectors for common regulatory elements, while fine-tuning adapts these detectors to recognize cell type-specific variations in enhancer-promoter architecture and transcription factor binding patterns. Consequently, we developed the EPI-DynFusion-best model to leverage this dual-nature learning process, which shows promise in improving the identification of cell line-specific enhancer-promoter interactions while maintaining robust performance on shared regulatory features. The specific training steps are as follows:

(1) Use EPI-DynFusion-gen as a pre-training model to utilize its generic feature learning capabilities to lay the foundation for subsequent steps.
(2) Retrain the model on the training set $D_{\text{train}}$ of each cell line so that the model can further learn cell line specific features and test it independently on the $D_{\text{test}}$ test set.
(3) Using AUROC and AUPR as evaluation metrics to assess model performance.

#### 5.8.1 Comparison of the three models

After employing the optimal training strategy, we evaluated the performance of the new model, as illustrated in Figure 10, and compared it with the other two models. The detailed performance metrics are provided in Table 8 and Table 9. The results show that the EPI-DynFusion-best model exhibits the best performance across multiple cell lines. This is due to the fact that the model uses a generic model as the pre-training base, which is able to capture shared features effectively. At the same time, by further training on each cell line, the model is equipped with the ability to recognize specific features. This dual advantage allows EPI-DynFusion-best to excel in recognizing both common features and cell line-specific features.

**TABLE 9:** Comparison of AUROC metrics for the three models.

Model/Cell lines	GM12878	HUVEC	HeLa-S3	IMR90	K562	NHEK
EPI-DynFusion-spe	0.932	0.947	**0.972**	0.910	0.940	0.987
EPI-DynFusion-gen	0.944	0.939	0.958	0.931	0.948	0.980
EPI-DynFusion-best	**0.954**	**0.962**	0.971	**0.935**	**0.958**	**0.989**

Experimental results demonstrate that our EPI-DynFusion-best model has achieved certain improvements across various performance indicators when compared to the baseline model (EPI-DynFusion-gen). In terms of the AUPR index, the model exhibited an enhancement of over 10% in all cell lines, with the most notable improvement observed in the HUVEC cell line (26.1%, increasing from 0.620 to 0.782), followed by the IMR90 cell line (21.8%, rising from 0.652 to 0.794) and the NHEK cell line (18.3%, increasing from 0.781 to 0.924). Regarding the AUROC index, despite the baseline model achieving a high performance level, the best model still demonstrated consistent improvement, with the HUVEC cell line showing a certain increase (2.4%, from 0.939 to 0.962). Notably, when compared to the specialized model (EPI-DynFusion-spe), the best model exhibited more stable performance across most cell lines, effectively mitigating the decline in AUROC metrics observed in some cell lines with the specialized model. This finding underscores the superiority of our training strategy in balancing model versatility and specificity.

#### 5.8.2 Performance comparison of the best EPI-DynFusion model with existing models

**FIGURE 11:**
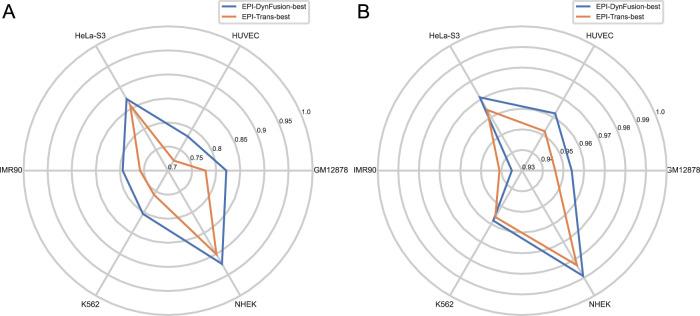
Comparison of AUPR and AUROC metrics for EPI-DynFusion model and EPI-Trans. **(A)** This figure presents a comparative analysis of AUPR values between the two models. **(B)** This figure presents a comparative analysis of AUROC values for the same models. The EPI-DynFusion-best model demonstrates superior performance across both metrics, exhibiting higher scores and greater distributional consistency, which indicates enhanced stability and predictive effectiveness.

To conduct an objective and rigorous performance evaluation, this study employed a series of standardized measures. First, the TargetFinder EPI dataset, which is widely recognized in the industry, was selected as the benchmark for evaluation. During the data processing phase, established standards were strictly adhered to, including a consistent method for dividing the training and test sets, as well as the use of fixed random seeds. Furthermore, a unified data enhancement strategy was implemented to address the issue of data imbalance. For the selection of evaluation metrics, this study utilized two widely accepted measures, AUROC and AUPR. Using these metrics, we conducted a comprehensive comparison of the optimal model, “EPI-DynFusion-best,” developed in this study against several mainstream models (e.g., EPI-Trans, EPI-Mind, EPI-DLMH, EPIVAN, SPEID, PEP-WORD, and SIMCNN). The performance data for the models being compared are sourced from the study published by Ni et al. (2022). As illustrated in Tables 10 and 11, the performance details of each model across different cell lines are presented comprehensively. Additionally, Figure 11A displays the comparative analysis of the models based on the AUPR indicator, while Figure 11B presents the comparative analysis based on the AUROC indicator.

**TABLE 10:** Comparison of AUPR metrics for existing models.

Model/cell lines	GM12878	HUVEC	HeLa-S3	IMR90	K562	NHEK	AVG
EPI-DynFusion-best	**0.821**	**0.782**	**0.872**	0.794	0.804	**0.924**	**0.833**
EPI-Trans-best	0.778	0.724	0.857	0.758	0.758	0.901	0.796
EPI-Mind-best	0.796	0.710	0.843	0.769	0.756	0.903	0.796
EPI-DLMH	0.819	0.720	0.824	0.818	0.826	0.893	0.817
EPIVAN	0.779	0.691	0.800	0.762	0.804	0.864	0.783
EPIHC	0.796	0.675	0.827	0.759	0.794	0.854	0.784
RAEPI	0.689	0.638	0.739	0.753	0.750	0.837	0.734
SPEID	0.773	0.523	0.797	0.732	0.771	0.852	0.741
PEP-WORD	0.807	0.760	0.803	**0.868**	**0.836**	0.880	0.826
SIMCNN	0.706	0.640	0.737	0.737	0.679	0.882	0.730

**TABLE 11:** Comparison of AUROC metrics for existing models.

Model/Cell lines	GM12878	HUVEC	HeLa-S3	IMR90	K562	NHEK	AVG
EPI-DynFusion-best	**0.954**	**0.962**	**0.971**	0.935	**0.958**	**0.989**	**0.962**
EPI-Trans-best	0.946	0.952	0.964	0.941	0.956	0.983	0.957
EPI-Mind-best	0.951	0.945	0.961	0.922	0.946	0.987	0.952
EPI-DLMH	0.949	0.948	0.952	0.948	0.955	0.977	0.955
EPIVAN	0.937	0.925	0.941	0.933	0.946	0.969	0.942
EPIHC	0.931	0.908	0.948	0.916	0.937	0.955	0.933
RAEPI	0.941	0.907	0.951	0.919	0.919	0.965	0.934
SPEID	0.916	0.904	0.923	0.915	0.922	0.950	0.922
PEP-WORD	0.842	0.845	0.843	0.898	0.883	0.917	0.871
SIMCNN	0.941	0.933	0.949	**0.951**	0.943	0.962	0.947

In terms of model performance evaluation, our EPI-DynFusion-best model demonstrated exceptional performance. Specifically, regarding AUROC metrics, the model achieved the highest results in five cell lines: GM12878, HUVEC, HeLa-S3, K562, and NHEK, with AUROC values of 0.954, 0.962, 0.971, 0.958, and 0.962, respectively. Notably, in the HeLa-S3 cell line, the improvement was certain compared to other models, reaching up to 12.8%. In the GM12878, HUVEC, K562, and NHEK cell lines, the enhancements were also substantial, at 11.2%, 11.7%, 7.5%, and 7.2%, respectively. Although the performance in the IMR90 cell line exhibited slight fluctuations, it remained consistently high overall and was only marginally lower than some benchmark models in individual comparisons.

EPI-DynFusion-best also demonstrated robust performance in the AUPR assessment index, achieving scores of 0.821, 0.782, 0.872, and 0.924 for the GM12878, HUVEC, HeLa-S3, and NHEK cell lines, respectively. This reflects enhancements of 11.5%, 25.9%, 17%, and 20% over competing models. For the IMR90 and K562 cell lines, the model maintained strong performance, albeit with slightly reduced effectiveness in certain comparisons.

Overall, EPI-DynFusion-best attained an average AUROC of 96.2% and an average AUPR of 83.3% across the six cell lines, outperforming all models considered in this study. These findings clearly indicate that EPI-DynFusion-best possesses certain advantages in predicting enhancer-promoter interactions, with overall performance better to most existing relevant models.

## 6 Motif analysis

To verify the biological interpretability of the laws learned by the EPI-DynFusion model and to identify the key sequence motifs on which its predictive decisions depend, we employed the *in silico* mutagenesis analysis strategy proposed in related studies. Specifically, we first screened samples from the training set that were predicted as positive by the model with high confidence (prediction probability >0.95) and conducted perturbation analysis on their enhancer and promoter sequences. We systematically perturbed the input sequences using a sliding window of 6 bp in length and a step size of 3 bp, masking the bases within the window and re-inputting them into the model for prediction. This allowed us to calculate the change in probability before and after the perturbation and to define their significance scores. This approach is analogous to the perturbation and cluster analysis strategy utilized in the study by Alex et al. (2024). The effect of perturbation position on prediction scores is illustrated in Supplementary Material S1: Supplementary Figures S1–S6.

After counting the high causal importance segments in all samples, we conducted an unsupervised cluster analysis on the candidate sequences. This was achieved by encoding the sequences using 3-mer frequency distribution features and applying a hierarchical clustering method (Euclidean distance combined with the Ward linkage strategy). Based on the number of segments in each cluster and their corresponding importance scores, we prioritized specific motif clusters for further analysis. Subsequently, we utilized the MEME tool (Trisha and Charles, 1994) for *de novo* motif discovery, focusing on the higher frequency dominant motifs and the top-ranked clusters. We then compared these motifs with known transcription factor binding sites from the JASPAR (Oriol et al., 2019) database using TOMTOM (Gupta et al., 2007). The results indicated that both the dominant motifs and those within the highly ranked clusters exhibited significant similarity (q-value <0.05) with multiple known transcription factor binding sites, thereby validating the advantages of EPI-DynFusion in identifying biologically significant regulatory patterns. The calculation of the cluster importance score and the significance score before and after sequence perturbation is shown in Supplementary Material S1: Formula (1.1).

**FIGURE 12:**
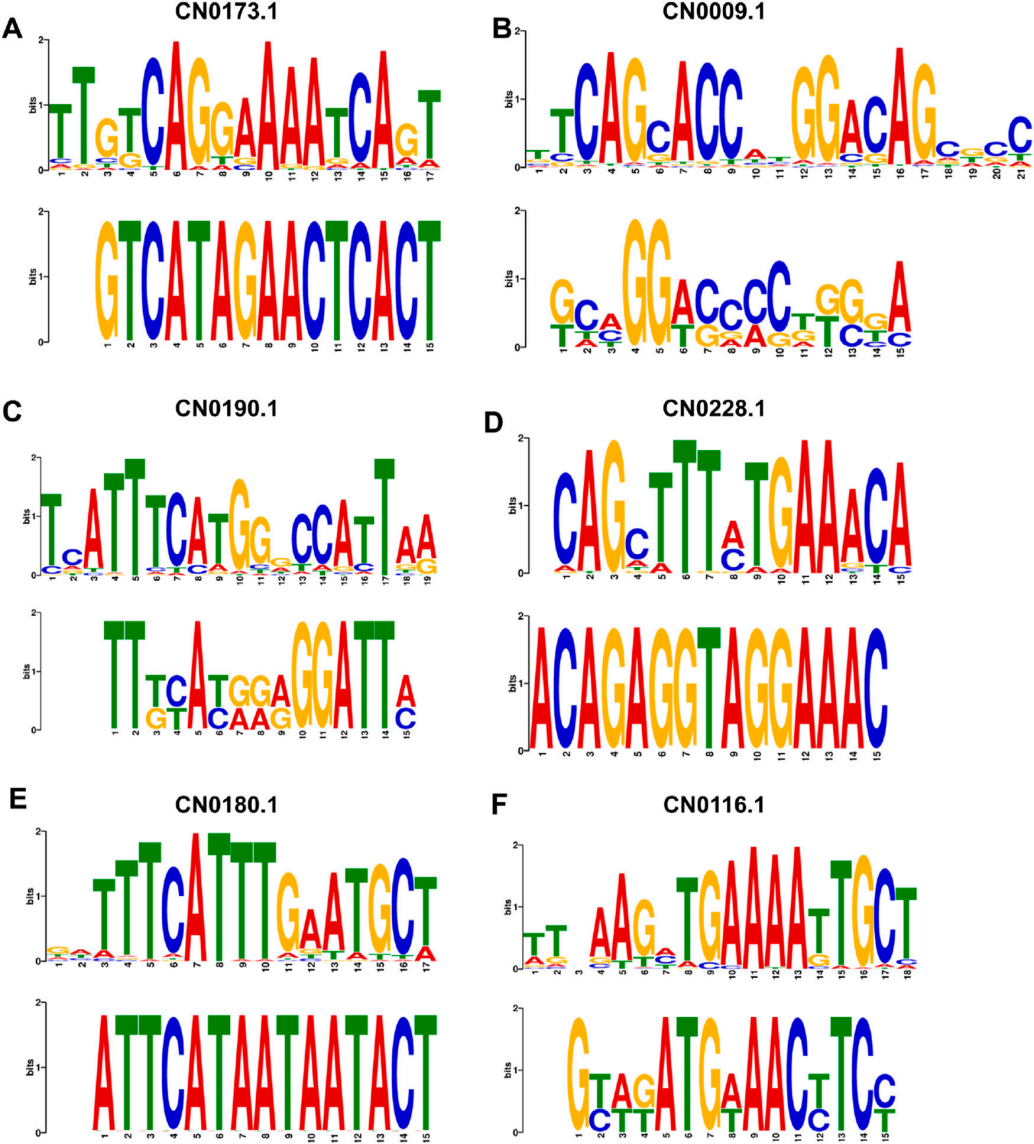
Visual display of mapping representative motifs to known transcription factor binding sites. **(A)** CN0173 matches motifs extracted from GM12878. **(B)** CN0009 matches motifs extracted from HeLa-S3. **(C)** CN0190 matched with the motifs extracted from IMR90 **(D)** CN0228 matched with the motifs extracted from HUVEC. **(E)** Base sequence match between CN0180 and K562 extracted. **(F)** CN0116 matches with the motifs extracted from NHEK.

Figure 12 presents representative examples of enhancer motif matches, including matches from the JASPAR database for CN0173, which features extracted motifs from the GM12878 cell line; CN0009, with motifs from the HeLa-S3 cell line; CN0190, with motifs from the IMR90 cell line; CN0228, with motifs from the HUVEC cell line; CN0180, corresponding to motifs from the K562 cell line; and CN0116, associated with motifs from the NHEK cell line. These transcription factors belong to distinct families and play critical roles in the regulation of gene expression. Specifically, CN0228 corresponds to the Sp1(Beishline et al., 2012) transcription factor, which regulates the expression of multiple genes primarily by binding to GC-rich sequences. CN0173 is linked to the NF-Y (Dolfini et al., 2025) family of transcription factors, which are involved in the regulation of the cell cycle, DNA repair, and metabolic genes. CN0190 is part of the E2F (Attwooll et al., 2004) family, which is extensively involved in cell cycle regulation, DNA replication, and cell proliferation. Furthermore, CN0009 is associated with the AP-1 (Wu et al., 2021) transcription factor complex, which is crucial for cell growth, differentiation, and stress response. CN0180 corresponds to the AP-2 (Yu et al., 2024) family of transcription factors, which is involved in embryonic development, cell differentiation, and immune function. Lastly, CN0116 belongs to the STAT (Takeda and Akira, 2000) family of transcription factors, playing a pivotal role in cytokine signaling and immune response. These matching results further confirm the effectiveness of EPI-DynFusion in capturing and characterizing genomic features, thereby enhancing our understanding of the mechanisms underlying enhancer-promoter interactions (EPIs). Detailed motif matching results can be found in Supplementary Material S2.

## 7 Performance of EPI-DynFusion on new datasets

**FIGURE 13:**
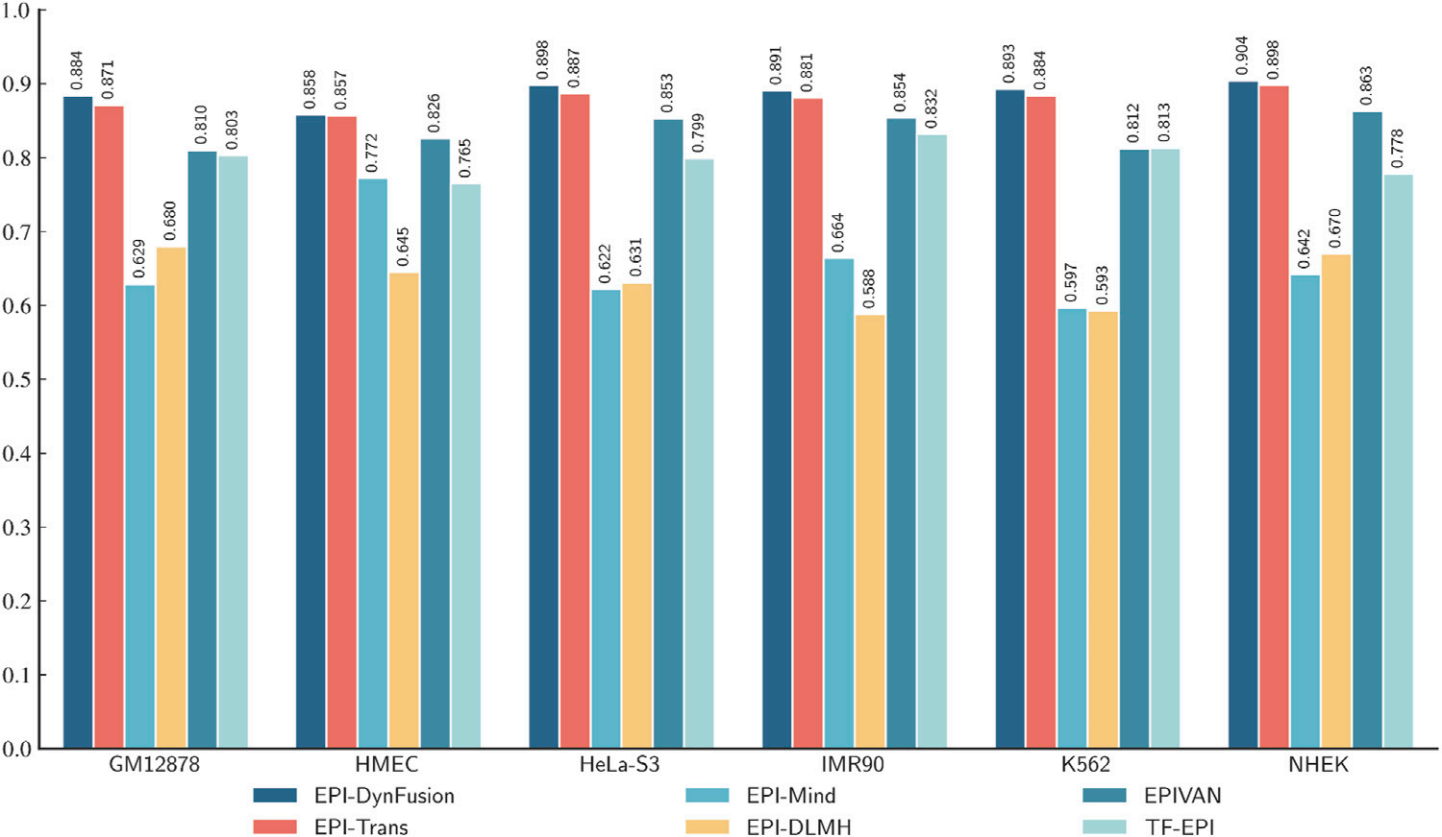
Values of AUROC compared with existing models on the dataset of BENGI. The analysis includes six human cell lines. This graph shows the AUROC values of different algorithms across multiple cell lines to evaluate the classification performance of the models under unbalanced data conditions. EPI-DynFusion demonstrated superior performance across most cell lines, achieving AUROC values close to or even exceeding 0.8. In contrast, EPI-Trans and EPI-DLMH showed poor performance in certain cell lines. Notably, the HeLa-S3 cell line consistently yielded higher AUROC values, particularly for EPI-DynFusion and EPI-Mind, indicating their enhanced generalization capabilities.

**FIGURE 14:**
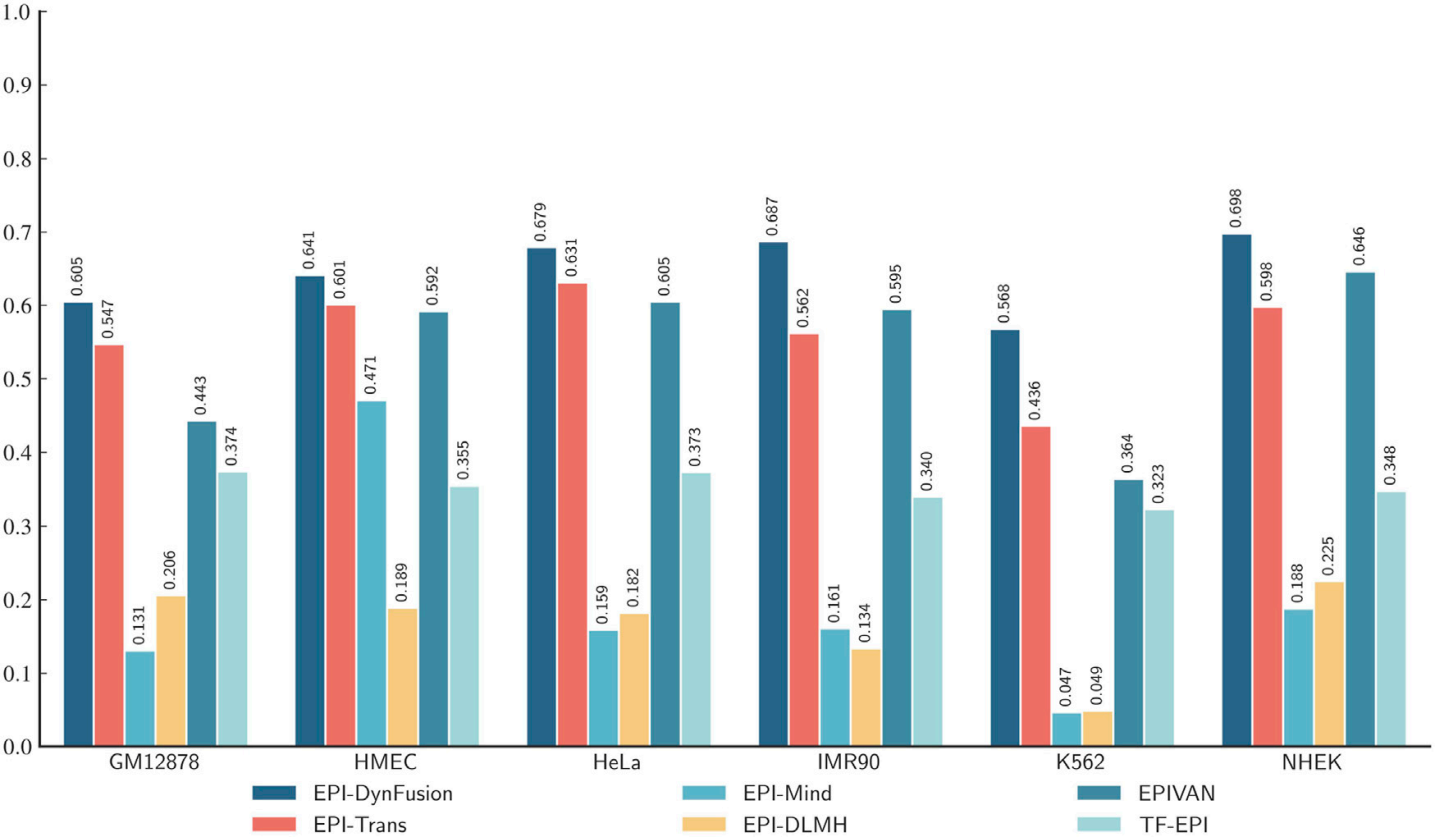
Values of AUPR compared with existing models on the dataset of BENGI. The analysis includes six human cell lines. This graph shows the AUPR values of different algorithms across multiple cell lines to evaluate the classification performance of the models under unbalanced data conditions. EPI‐DynFusion performs best on most cell lines with the highest AUPR values. In contrast, EPI‐Trans and EPIVAN perform poorly on some cell lines, while EPI‐Mind shows slightly erratic performance across certain cell types. Notably, the NHEK cell line displays more balanced AUPR values across different methods.

To further validate the performance of our model, we evaluated the EPI-DynFusion model using the BENGI dataset (Liu et al., 2024), which comprises 45,182 positive samples and 307,135 negative samples. These samples were randomly divided into a training set and an independent test set in a 9:1 ratio, as detailed in Table 1 of Supplementary Material S1. It is important to note that we re-trained and tested all comparative models utilizing the publicly available code provided by Chen et al. along with the hyperparameter settings reported in their paper. As illustrated in Figures 13, 14, our model outperforms the other four predictive models across various cell lines. EPI-DynFusion exhibits significant improvements in both AUROC and AUPR metrics, demonstrating exceptional robustness. Compared to the next best model, EPI-Trans, the AUROC improvement ranges from 0.1% to 1.3%, while the enhancement in AUPR is even more pronounced, with an increase of 4.0%–13.2%. Additionally, EPI-DynFusion displayed significant advantages over the less effective models. For instance, in the K562 cell line, the AUPR increased from 0.047 in EPI-Mind to 0.568 in EPI-DynFusion, marking a 52.1% increase. Notably, EPI-DynFusion demonstrated consistent performance enhancement across all cell lines, with both AUROC and AUPR metrics remaining high and stable, thereby showcasing its strong robustness in capturing complex epigenetic features across diverse cell lines.

In addition, we compared our results with those of the TF-EPI model, whose performance metrics were obtained from the data presented in its original publication. According to the findings reported in the TF-EPI paper, the model achieved a maximum score of 83.4% on the AUROC metric, while its AUPR score was 37.3%. In contrast, EPI-DynFusion demonstrated an improvement of 7.0% on AUROC and up to 22.5% on AUPR compared to TF-EPI. These results further underscore the superiority and enhanced robustness of EPI-DynFusion in capturing complex epigenetic features and in predicting enhancer-promoter interactions.

## 8 Conclusion

In this paper, we present an innovative deep learning model, named EPI-DynFusion, designed to automatically detect and predict enhancer-promoter interactions. The model comprises three components: sequence embedding, framework of EPI-DynFusion model, and EPI prediction. During data processing, DNA sequences of enhancers and promoters are first input into the model, where these biological sequences are transformed into a high-dimensional feature matrix using the pre-trained dna2vec technique. Next, a two-layer CNN network structure is employed to accurately extract local features, effectively capturing short-range spatial patterns and local feature information in DNA sequences. Subsequently, we introduce the Transformer encoder mechanism to capture long-range dependencies between sequences and global contextual information.

Unlike existing methods that rely on fixed-weight combination or simple concatenation of features, our model implements a novel dynamic feature fusion strategy. This strategy intelligently integrates Transformer and BIGRU features through a dual-stream adaptive weighting mechanism. Specifically, our model learns context-aware dynamic weights that automatically adjust based on the intrinsic characteristics of each sequence pair, enabling more nuanced feature emphasis. This adaptive fusion is further enhanced by the CBAM attention module, which refines both channel and spatial feature representations. Through this sophisticated fusion approach, our model can effectively identify and emphasize the most discriminative features for each specific sequence context, leading to high-precision EPI prediction results in the final prediction layer.

Experimental results show that EPI-DynFusion shows good performance in a variety of cell lines, and its prediction accuracy is better than existing mainstream methods. Furthermore, the model allows for fine-tuning on specific cell line data, enabling further optimization of performance while maintaining common features across cell lines and accurately capturing the unique characteristics of individual cell lines. This capability, combined with our innovative dynamic feature fusion mechanism, positions EPI-DynFusion as a robust and advanced analytical tool for genomic research.

It is worth noting that the use of pre-trained DNA embeddings, such as dna2vec, may affect the model’s ability to process new sequences or datasets. Additionally, our current evaluation primarily relies on static benchmark datasets, such as those provided by TargetFinder, which offer valuable observational insights for EPI but still have room for improvement in characterizing the dynamic nature of chromatin interactions and gene regulation. Under the LOCO cross-validation framework, the performance of all models decreased compared to random data partitions, confirming that cross-chromosomal generalization is an inherent challenge for current methods. Although EPI-DynFusion performs exceptionally well across many cell lines, this also indicates that the capability for cross-chromosomal generalization remains a critical factor influencing model performance in practical applications.

In future work, we plan to enhance the model’s capabilities in several key areas. First, to reduce reliance on fixed pre-trained vectors, such as dna2vec, we will explore more flexible and context-aware sequence representation methods. This includes introducing task-driven embedding mechanisms that enable the model to adaptively learn feature representations throughout the training process. Second, to accurately represent the dynamic characteristics of chromatin structure, we will integrate time-series chromatin accessibility data, such as ATAC-seq or Hi-C data collected at different time points or under various stimuli, to capture the dynamic changes in EPI. Additionally, to further address the issue of cross-chromosomal generalization, we will explore incorporating richer genomic context, three-dimensional chromatin structure, or cross-chromosomal graph learning methods to enhance the model’s adaptability and generalization capabilities across different chromosomes. Through these enhancements, we anticipate that EPI-DynFusion will make a more significant contribution to broader biological research applications. Through these enhancements, we anticipate that EPI-DynFusion will make a more significant contribution to broader biological research applications.

## Data Availability

Publicly available datasets were analyzed in this study. The motif matching data can be found in the JASPAR 2020 database here: (https://jaspar2020.genereg.net/). The code and benchmark datasets for the EPI-DynFusion model can be accessed here: https://github.com/zhangao-clude/EPI-DynFusion.

## Abbreviations

EPI, Enhancer-promoter interaction; CBAM, Convolutional Block Attention Module; GRU, Gated recurrent unit; BiGRU, Bidirectional Gated Recurrent Unit; CNN, Convolutional neural network; LSTM, Long short-term memory; RNN, Recurrent neural network; AUROC, Area under receiver operating characteristic curve; AUPR, Area under precision-recall curve; ROC, Receiver Operating Characteristic Curve; ChIA-PET, Chromatin Interaction Analysis by Paired-End Tag Sequencing.
