# Supplementary material for: EPI-DynFusion: enhancer-promoter interaction prediction model based on sequence features and dynamic fusion mechanisms
Source: Front Genet. 2025 Jul 23;16:1614222. doi: 10.3389/fgene.2025.1614222 (PMC12325019; doi:10.3389/fgene.2025.1614222)
Supplement: Supplementary file 2 [file DataSheet2.docx]

Supplementary Material

# Supplementary Tables

**Table 1 The benchmark dataset of BENGI**

| Cell Lines | Pos Samples | Neg Samples |
| --- | --- | --- |
| GM12878 | 32,497 | 152,910 |
| HMEC | 2286 | 20,019 |
| HeLa-S3 | 4346 | 34,057 |
| IMR90 | 1468 | 13,268 |
| K562 | 2765 | 73,299 |
| NHEK | 1820 | 13,582 |
| Total | 45182 | 307135 |

**Table 2 23-fold cross-validation of EPI-DynFusion in LOCO Split setup under different cell lines**

| Left Out Chromosome | GM12878 | HeLa | HUVEC | IMR90 | K562 | NHEK |
| --- | --- | --- | --- | --- | --- | --- |
| chr1 | 0.580 | 0.590 | 0.558 | 0.583 | 0.578 | 0.617 |
| chr2 | 0.585 | 0.588 | 0.671 | 0.673 | 0.615 | 0.650 |
| chr3 | 0.602 | 0.643 | 0.596 | 0.637 | 0.602 | 0.665 |
| chr4 | 0.591 | 0.746 | 0.693 | 0.571 | 0.808 | 0.614 |
| chr5 | 0.667 | 0.663 | 0.665 | 0.680 | 0.653 | 0.621 |
| chr6 | 0.587 | 0.608 | 0.602 | 0.670 | 0.565 | 0.577 |
| chr7 | 0.612 | 0.686 | 0.688 | 0.624 | 0.646 | 0.692 |
| chr8 | 0.591 | 0.663 | 0.652 | 0.618 | 0.693 | 0.691 |
| chr9 | 0.612 | 0.591 | 0.585 | 0.673 | 0.555 | 0.653 |
| chr10 | 0.650 | 0.694 | 0.737 | 0.671 | 0.675 | 0.642 |
| chr11 | 0.653 | 0.615 | 0.591 | 0.713 | 0.582 | 0.611 |
| chr12 | 0.610 | 0.555 | 0.660 | 0.680 | 0.570 | 0.623 |
| chr13 | 0.584 | 0.740 | 0.692 | 0.663 | 0.762 | 0.756 |
| chr14 | 0.591 | 0.651 | 0.642 | 0.669 | 0.776 | 0.641 |
| chr15 | 0.706 | 0.677 | 0.676 | 0.790 | 0.652 | 0.592 |
| chr16 | 0.617 | 0.65 | 0.652 | 0.716 | 0.631 | 0.590 |
| chr17 | 0.579 | 0.617 | 0.552 | 0.660 | 0.578 | 0.640 |
| chr18 | 0.549 | 0.689 | 0.660 | 0.593 | 0.730 | 0.729 |
| chr19 | 0.568 | 0.616 | 0.689 | 0.514 | 0.608 | 0.714 |
| chr20 | 0.701 | 0.728 | 0.631 | 0.633 | 0.662 | 0.653 |
| chr21 | 0.674 | 0.735 | 0.764 | 0.932 | 0.714 | 0.601 |
| chr22 | 0.568 | 0.662 | 0.618 | 0.780 | 0.635 | 0.632 |
| chr23 | 0.667 | 0.722 | 0.609 | 0.717 | 0.662 | 0.739 |

**Table3 23-fold cross-validation of EPI-Trans in LOCO Split setup under different cell lines**

| Left Out Chromosome | GM12878 | HeLa-S3 | HUVEC | IMR90 | K562 | NHEK |
| --- | --- | --- | --- | --- | --- | --- |
| chr1 | 0.552 | 0.580 | 0.587 | 0.579 | 0.583 | 0.515 |
| chr2 | 0.610 | 0.662 | 0.714 | 0.676 | 0.565 | 0.625 |
| chr3 | 0.634 | 0.745 | 0.554 | 0.721 | 0.555 | 0.601 |
| chr4 | 0.577 | 0.669 | 0.732 | 0.577 | 0.494 | 0.687 |
| chr5 | 0.696 | 0.683 | 0.666 | 0.582 | 0.580 | 0.545 |
| chr6 | 0.584 | 0.650 | 0.521 | 0.601 | 0.552 | 0.556 |
| chr7 | 0.580 | 0.637 | 0.705 | 0.603 | 0.631 | 0.657 |
| chr8 | 0.619 | 0.700 | 0.684 | 0.613 | 0.624 | 0.742 |
| chr9 | 0.642 | 0.635 | 0.541 | 0.715 | 0.562 | 0.621 |
| chr10 | 0.543 | 0.676 | 0.686 | 0.63 | 0.584 | 0.591 |
| chr11 | 0.614 | 0.672 | 0.572 | 0.672 | 0.595 | 0.561 |
| chr12 | 0.599 | 0.630 | 0.686 | 0.682 | 0.598 | 0.571 |
| chr13 | 0.582 | 0.742 | 0.709 | 0.465 | 0.583 | 0.743 |
| chr14 | 0.637 | 0.628 | 0.617 | 0.637 | 0.596 | 0.498 |
| chr15 | 0.635 | 0.654 | 0.607 | 0.701 | 0.625 | 0.586 |
| chr16 | 0.639 | 0.674 | 0.624 | 0.680 | 0.609 | 0.574 |
| chr17 | 0.636 | 0.553 | 0.492 | 0.590 | 0.605 | 0.636 |
| chr18 | 0.534 | 0.590 | 0.671 | 0.695 | 0.720 | 0.651 |
| chr19 | 0.597 | 0.543 | 0.555 | 0.371 | 0.531 | 0.673 |
| chr20 | 0.634 | 0.616 | 0.669 | 0.585 | 0.640 | 0.627 |
| chr21 | 0.737 | 0.737 | 0.786 | 0.738 | 0.886 | 0.617 |
| chr22 | 0.529 | 0.747 | 0.622 | 0.692 | 0.618 | 0.612 |
| chr23 | 0.599 | 0.636 | 0.633 | 0.743 | 0.649 | 0.671 |

**Table 4 23-fold cross-validation of EPI-Mind in LOCO Split setup under different cell lines**

| Left Out Chromosome | GM12878 | HeLa | HUVEC | IMR90 | K562 | NHEK |
| --- | --- | --- | --- | --- | --- | --- |
| chr1 | 0.523 | 0.513 | 0.537 | 0.586 | 0.552 | 0.509 |
| chr2 | 0.564 | 0.518 | 0.579 | 0.414 | 0.511 | 0.610 |
| chr3 | 0.591 | 0.607 | 0.576 | 0.57 | 0.572 | 0.587 |
| chr4 | 0.603 | 0.756 | 0.598 | 0.655 | 0.591 | 0.515 |
| chr5 | 0.599 | 0.553 | 0.551 | 0.583 | 0.555 | 0.582 |
| chr6 | 0.518 | 0.594 | 0.543 | 0.539 | 0.546 | 0.567 |
| chr7 | 0.631 | 0.584 | 0.670 | 0.621 | 0.643 | 0.668 |
| chr8 | 0.549 | 0.580 | 0.594 | 0.529 | 0.705 | 0.603 |
| chr9 | 0.592 | 0.573 | 0.608 | 0.586 | 0.502 | 0.640 |
| chr10 | 0.563 | 0.600 | 0.622 | 0.621 | 0.576 | 0.533 |
| chr11 | 0.663 | 0.574 | 0.586 | 0.553 | 0.548 | 0.531 |
| chr12 | 0.603 | 0.536 | 0.586 | 0.539 | 0.568 | 0.624 |
| chr13 | 0.614 | 0.646 | 0.618 | 0.667 | 0.642 | 0.699 |
| chr14 | 0.590 | 0.552 | 0.568 | 0.541 | 0.629 | 0.714 |
| chr15 | 0.548 | 0.667 | 0.528 | 0.531 | 0.581 | 0.590 |
| chr16 | 0.589 | 0.598 | 0.566 | 0.516 | 0.549 | 0.601 |
| chr17 | 0.553 | 0.609 | 0.609 | 0.552 | 0.596 | 0.640 |
| chr18 | 0.550 | 0.600 | 0.606 | 0.643 | 0.501 | 0.576 |
| chr19 | 0.554 | 0.584 | 0.567 | 0.615 | 0.539 | 0.658 |
| chr20 | 0.556 | 0.611 | 0.601 | 0.607 | 0.506 | 0.668 |
| chr21 | 0.750 | 0.632 | 0.774 | 0.954 | 0.780 | 0.544 |
| chr22 | 0.630 | 0.680 | 0.607 | 0.678 | 0.576 | 0.610 |
| chr23 | 0.554 | 0.677 | 0.736 | 0.767 | 0.560 | 0.527 |

**Table 5 23-fold cross-validation of EPI-DLMH in LOCO Split setup under different cell lines**

| Left Out Chromosome | GM12878 | HeLa | HUVEC | IMR90 | K562 | NHEK |
| --- | --- | --- | --- | --- | --- | --- |
| chr1 | 0.572 | 0.502 | 0.571 | 0.588 | 0.568 | 0.530 |
| chr2 | 0.621 | 0.598 | 0.649 | 0.621 | 0.582 | 0.552 |
| chr3 | 0.502 | 0.596 | 0.579 | 0.525 | 0.570 | 0.545 |
| chr4 | 0.598 | 0.789 | 0.603 | 0.656 | 0.604 | 0.729 |
| chr5 | 0.637 | 0.616 | 0.625 | 0.556 | 0.658 | 0.679 |
| chr6 | 0.642 | 0.658 | 0.593 | 0.555 | 0.549 | 0.513 |
| chr7 | 0.591 | 0.589 | 0.620 | 0.574 | 0.619 | 0.516 |
| chr8 | 0.632 | 0.633 | 0.672 | 0.620 | 0.724 | 0.588 |
| chr9 | 0.617 | 0.637 | 0.590 | 0.617 | 0.551 | 0.612 |
| chr10 | 0.537 | 0.672 | 0.683 | 0.503 | 0.627 | 0.626 |
| chr11 | 0.566 | 0.672 | 0.622 | 0.546 | 0.624 | 0.657 |
| chr12 | 0.591 | 0.563 | 0.621 | 0.651 | 0.562 | 0.638 |
| chr13 | 0.530 | 0.823 | 0.598 | 0.685 | 0.428 | 0.629 |
| chr14 | 0.584 | 0.529 | 0.485 | 0.608 | 0.595 | 0.543 |
| chr15 | 0.610 | 0.643 | 0.588 | 0.704 | 0.657 | 0.683 |
| chr16 | 0.577 | 0.675 | 0.606 | 0.629 | 0.655 | 0.619 |
| chr17 | 0.532 | 0.620 | 0.576 | 0.647 | 0.590 | 0.574 |
| chr18 | 0.582 | 0.798 | 0.664 | 0.621 | 0.670 | 0.379 |
| chr19 | 0.573 | 0.604 | 0.498 | 0.611 | 0.559 | 0.455 |
| chr20 | 0.635 | 0.678 | 0.568 | 0.464 | 0.648 | 0.714 |
| chr21 | 0.647 | 0.563 | 0.737 | 0.553 | 0.614 | 0.609 |
| chr22 | 0.613 | 0.577 | 0.627 | 0.525 | 0.529 | 0.585 |
| chr23 | 0.566 | 0.713 | 0.696 | 0.550 | 0.594 | 0.729 |

**Table 6 23-fold cross-validation of EPIVAN in LOCO Split setup under different cell lines**

| Left Out Chromosome | GM12878 | HeLa | HUVEC | IMR90 | K562 | NHEK |
| --- | --- | --- | --- | --- | --- | --- |
| chr1 | 0.629 | 0.559 | 0.595 | 0.520 | 0.585 | 0.485 |
| chr2 | 0.616 | 0.511 | 0.648 | 0.580 | 0.616 | 0.660 |
| chr3 | 0.523 | 0.675 | 0.524 | 0.572 | 0.588 | 0.619 |
| chr4 | 0.553 | 0.670 | 0.686 | 0.488 | 0.588 | 0.564 |
| chr5 | 0.679 | 0.589 | 0.650 | 0.591 | 0.589 | 0.497 |
| chr6 | 0.571 | 0.602 | 0.577 | 0.484 | 0.566 | 0.525 |
| chr7 | 0.595 | 0.525 | 0.623 | 0.582 | 0.560 | 0.688 |
| chr8 | 0.598 | 0.718 | 0.591 | 0.678 | 0.507 | 0.513 |
| chr9 | 0.560 | 0.538 | 0.566 | 0.621 | 0.671 | 0.674 |
| chr10 | 0.573 | 0.666 | 0.659 | 0.612 | 0.614 | 0.566 |
| chr11 | 0.633 | 0.531 | 0.595 | 0.660 | 0.620 | 0.603 |
| chr12 | 0.578 | 0.525 | 0.654 | 0.628 | 0.573 | 0.609 |
| chr13 | 0.570 | 0.675 | 0.718 | 0.545 | 0.573 | 0.639 |
| chr14 | 0.580 | 0.703 | 0.592 | 0.670 | 0.556 | 0.331 |
| chr15 | 0.615 | 0.671 | 0.619 | 0.636 | 0.525 | 0.565 |
| chr16 | 0.637 | 0.760 | 0.523 | 0.596 | 0.608 | 0.604 |
| chr17 | 0.599 | 0.562 | 0.531 | 0.564 | 0.507 | 0.521 |
| chr18 | 0.557 | 0.499 | 0.521 | 0.572 | 0.387 | 0.576 |
| chr19 | 0.566 | 0.589 | 0.539 | 0.603 | 0.577 | 0.454 |
| chr20 | 0.567 | 0.694 | 0.591 | 0.459 | 0.600 | 0.629 |
| chr21 | 0.567 | 0.563 | 0.693 | 0.509 | 0.568 | 0.609 |
| chr22 | 0.659 | 0.813 | 0.421 | 0.579 | 0.516 | 0.471 |
| chr23 | 0.512 | 0.722 | 0.680 | 0.815 | 0.483 | 0.501 |

**Table 7 23-fold cross-validation of LOCO-EPI in LOCO Split setup under different cell lines**

| Left Out Chromosome | GM12878 | HeLa-S3 | HUVEC | IMR90 | K562 | NHEK |
| --- | --- | --- | --- | --- | --- | --- |
| Chr1 | 0.575 | 0.604 | 0.591 | 0.544 | 0.559 | 0.576 |
| Chr2 | 0.613 | 0.540 | 0.687 | 0.692 | 0.589 | 0.592 |
| Chr3 | 0.609 | 0.604 | 0.595 | 0.594 | 0.532 | 0.634 |
| Chr4 | 0.522 | 0.455 | 0.672 | 0.600 | 0.580 | 0.608 |
| Chr5 | 0.703 | 0.64 | 0.614 | 0.668 | 0.475 | 0.785 |
| Chr6 | 0.531 | 0.581 | 0.532 | 0.705 | 0.544 | 0.582 |
| Chr7 | 0.476 | 0.627 | 0.681 | 0.578 | 0.615 | 0.709 |
| Chr8 | 0.584 | 0.710 | 0.615 | 0.655 | 0.543 | 0.621 |
| Chr9 | 0.598 | 0.560 | 0.589 | 0.665 | 0.509 | 0.599 |
| Chr10 | 0.546 | 0.684 | 0.645 | 0.655 | 0.582 | 0.536 |
| Chr11 | 0.655 | 0.686 | 0.563 | 0.713 | 0.533 | 0.527 |
| Chr12 | 0.584 | 0.557 | 0.609 | 0.693 | 0.493 | 0.536 |
| Chr13 | 0.543 | 0.887 | 0.485 | 0.551 | 0.540 | 0.565 |
| Chr14 | 0.603 | 0.635 | 0.621 | 0.673 | 0.454 | 0.475 |
| Chr15 | 0.553 | 0.476 | 0.604 | 0.603 | 0.663 | 0.441 |
| Chr16 | 0.685 | 0.619 | 0.536 | 0.591 | 0.505 | 0.561 |
| Chr17 | 0.563 | 0.551 | 0.495 | 0.550 | 0.530 | 0.619 |
| Chr18 | 0.511 | 0.401 | 0.544 | 0.538 | 0.581 | 0.61 |
| Chr19 | 0.552 | 0.616 | 0.437 | 0.499 | 0.499 | 0.555 |
| Chr20 | 0.59 | 0.631 | 0.577 | 0.538 | 0.635 | 0.626 |
| Chr21 | 0.518 | 0.639 | 0.849 | 0.156 | 0.957 | 0.419 |
| Chr22 | 0.493 | 0.764 | 0.378 | 0.588 | 0.672 | 0.526 |
| Chr23 | 0.434 | 0.327 | 0.63 | 0.696 | 0.538 | 0.571 |

**Table 8 Performance of EPI-DynFusion model under different optimizers for AUROC in six cell lines**

| Optimizer/ cell lines | GM12878 | HUVEC | HeLa-S3 | IMR90 | K562 | NHEK |
| --- | --- | --- | --- | --- | --- | --- |
| Adam | 0.929 | 0.945 | **0.972** | 0.907 | 0.947 | 0.987 |
| RMSprop | 0.930 | 0.930 | 0.971 | 0.904 | **0.949** | **0.989** |
| NAdam | **0.932** | **0.947** | **0.972** | **0.910** | 0.940 | 0.987 |

**Table 9 Performance of EPI-DynFusion model under different optimizers for AUPR in six cell lines**

| Optimizer/ cell lines | GM12878 | HUVEC | HeLa-S3 | IMR90 | K562 | NHEK |
| --- | --- | --- | --- | --- | --- | --- |
| Adam | 0.783 | 0.685 | 0.852 | 0.724 | 0.774 | 0.889 |
| RMSprop | 0.711 | 0.685 | 0.867 | 0.696 | 0.776 | **0.942** |
| NAdam | **0.803** | **0.744** | **0.868** | **0.741** | **0.788** | 0.930 |

**Table 10 Performance of EPI-DynFusion model of AUROC under different enhancement magnification in six cell lines**

| Oversampe/  cell lines | GM12878 | HUVEC | HeLa-S3 | IMR90 | K562 | NHEK |
| --- | --- | --- | --- | --- | --- | --- |
| 0x | 0.919 | 0.903 | 0.943 | 0.876 | 0.910 | 0.970 |
| 5x | **0.939** | 0.943 | 0.972 | 0.898 | 0.939 | 0.979 |
| 10x | 0.921 | 0.933 | 0.963 | 0.911 | 0.938 | **0.990** |
| 15x | 0.926 | 0.928 | 0.958 | 0.912 | 0.935 | 0.987 |
| 20x | 0.932 | **0.947** | **0.972** | 0.910 | **0.940** | 0.987 |
| 25x | 0.929 | 0.938 | 0.964 | **0.915** | 0.938 | 0.987 |

**Table 11 Performance of EPI-DynFusion model of AUPR under different enhancement magnification in six cell lines**

| Oversampe/  cell lines | GM12878 | HUVEC | HeLa-S3 | IMR90 | K562 | NHEK |
| --- | --- | --- | --- | --- | --- | --- |
| 0x | 0.708 | 0.487 | 0.659 | 0.459 | 0.656 | 0.893 |
| 5x | 0.760 | 0.722 | 0.845 | 0.734 | 0.774 | 0.791 |
| 10x | 0.785 | 0.712 | 0.862 | 0.719 | 0.781 | **0.938** |
| 15x | 0.772 | 0.713 | 0.749 | 0.695 | 0.783 | 0.921 |
| 20x | **0.803** | **0.744** | **0.868** | 0.741 | **0.788** | 0.930 |
| 25x | 0.765 | 0.699 | 0.864 | **0.748** | 0.718 | 0.908 |

# **Table 12 The training time for all samples in minutes of the EPI-DynFusion models for the six cell lines**

| Model/Cell line | GM12878 | HeLa-S3 | HUVEC | IMR90 | K562 | NHEK | AVG |
| --- | --- | --- | --- | --- | --- | --- | --- |
| EPI-DynFusion-spe | 42.5 | 35 | 30.5 | 25.0 | 40.0 | 26.0 | 33.2 |
| EPI-DynFusion-gen | **-** | **-** | **-** | **-** | **-** | **-** | 200.0 |
| EPI-DynFusion-best | 41.5 | 34.5 | 30.0 | 25.0 | 39.5 | 25.5 | 32.7 |

# **Table 13 Training time (in minutes) for the EPI-DynFusion model on all samples on the six cell lines compared to the baseline model**

| Model/Cell line | GM12878 | HeLa-S3 | HUVEC | IMR90 | K562 | NHEK | AVG |
| --- | --- | --- | --- | --- | --- | --- | --- |
| EPI-DynFusion | 42.5 | 35.0 | 30.5 | 25.0 | 40.0 | 26.0 | 33.2 |
| EPI-Trans | 45.0 | 37.0 | 32.5 | 26.5 | 42.0 | 27.0 | 35.0 |
| EPI-Mind | 30.5 | 25.0 | 22.0 | 18.0 | 28.5 | 18.5 | 23.8 |
| EPI-DLMH | 18.5 | 15.5 | 13.5 | 11.0 | 17.5 | 11.5 | 14.6 |
| EPI-VAN | 15.0 | 12.5 | 11.0 | 9.0 | 14.5 | 9.0 | 11.8 |

# **Table 14 Comparison of Memory Occupancy of Transformer Modules and BiGRU Modules in EPI-DynFusion (in MB)**

| Model/Cell line | GM12878 | HeLa-S3 | HUVEC | IMR90 | K562 | NHEK | AVG |
| --- | --- | --- | --- | --- | --- | --- | --- |
| CNN | 4354.21 | 3843.74 | 3544.70 | 3173.25 | 4168.91 | 3222.59 | 3717.90 |
| CNN+BiGRU | 4356.03 | 3846.21 | 3545.51 | 3174.01 | 4169.11 | 3220.58 | 3718.58 |
| CNN+Transformer | 5651.44 | 5136.85 | 4838.46 | 4468.94 | 5464.09 | 4515.23 | 5012.50 |

# **Table 15 Comparison of memory occupied by EPI-DynFusion vs. baseline model during training (in MB)**

| Model/Cell line | GM12878 | HeLa-S3 | HUVEC | IMR90 | K562 | NHEK | AVG |
| --- | --- | --- | --- | --- | --- | --- | --- |
| EPI-DynFusion | 5547.84 | 5138.01 | 4840.63 | 4469.36 | 5464.39 | 4514.07 | 4995.72 |
| EPI-Trans | 4203.06 | 3796.00 | 3496.16 | 3125.58 | 4122.25 | 3172.09 | 3652.52 |
| EPI-Mind | 4966.08 | 4457.28 | 4157.73 | 3786.16 | 4784.85 | 3830.43 | 4330.42 |
| EPI-DLMH | 4232.67 | 3720.78 | 3432.67 | 3049.89 | 4047.25 | 3096.34 | 3596.60 |
| EPI-VAN | 4284.58 | 3778.83 | 3473.92 | 3101.33 | 4099.15 | 3150.21 | 3648.00 |

# Supplementary Figures

**
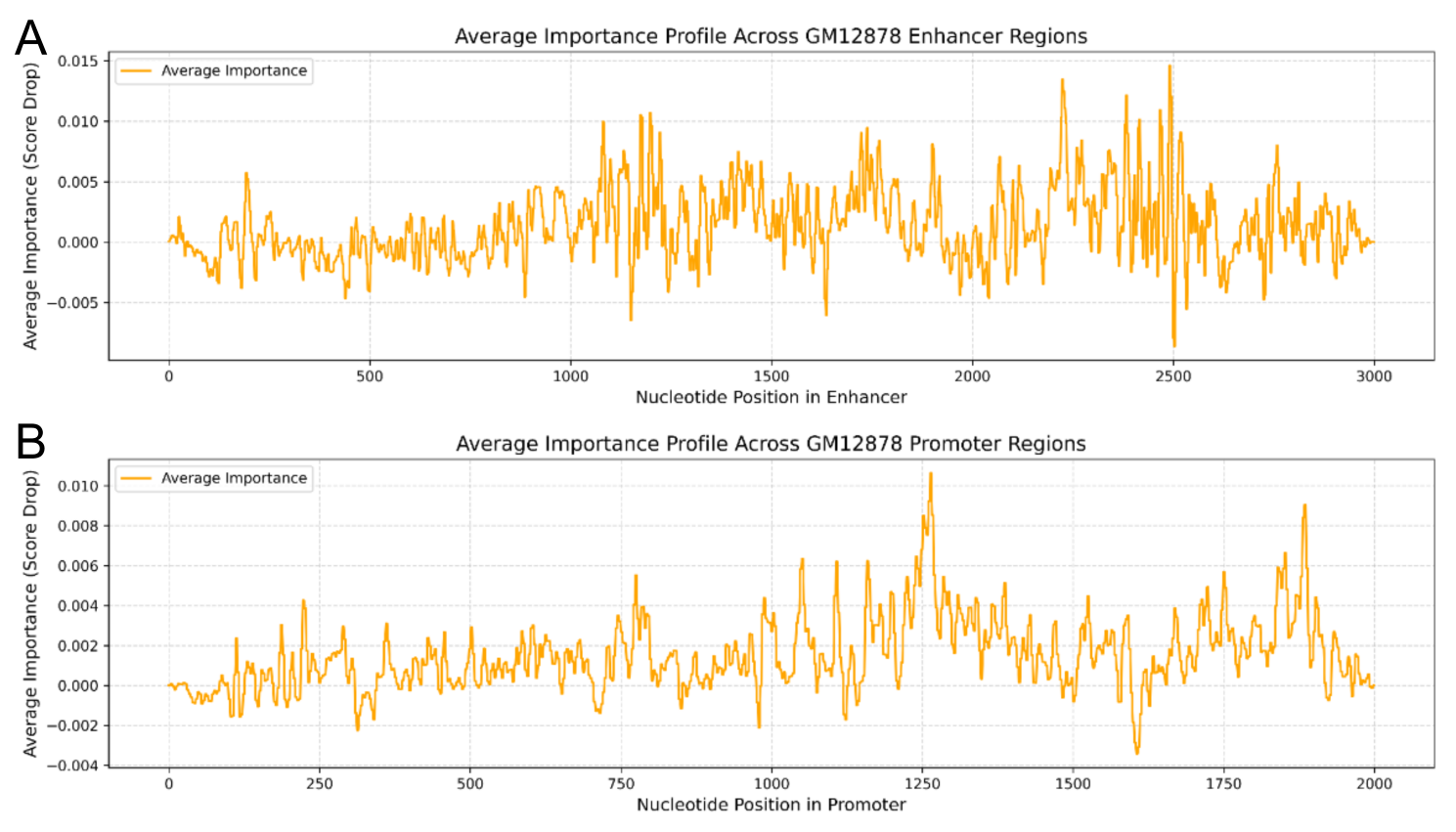
**

**Figure. 1 Effect of perturbation at different positions of GM12878 on the predicted scores** (A) Perturbation at GM12878 enhancer (B) Perturbation at GM12878 promoter. Where, the x-axis represents different positions on the DNA sequence, and the y-axis represents the score of the effect of perturbing the position on the predicted value, with higher peaks indicating that perturbing the position has a greater effect on the predicted score.

**
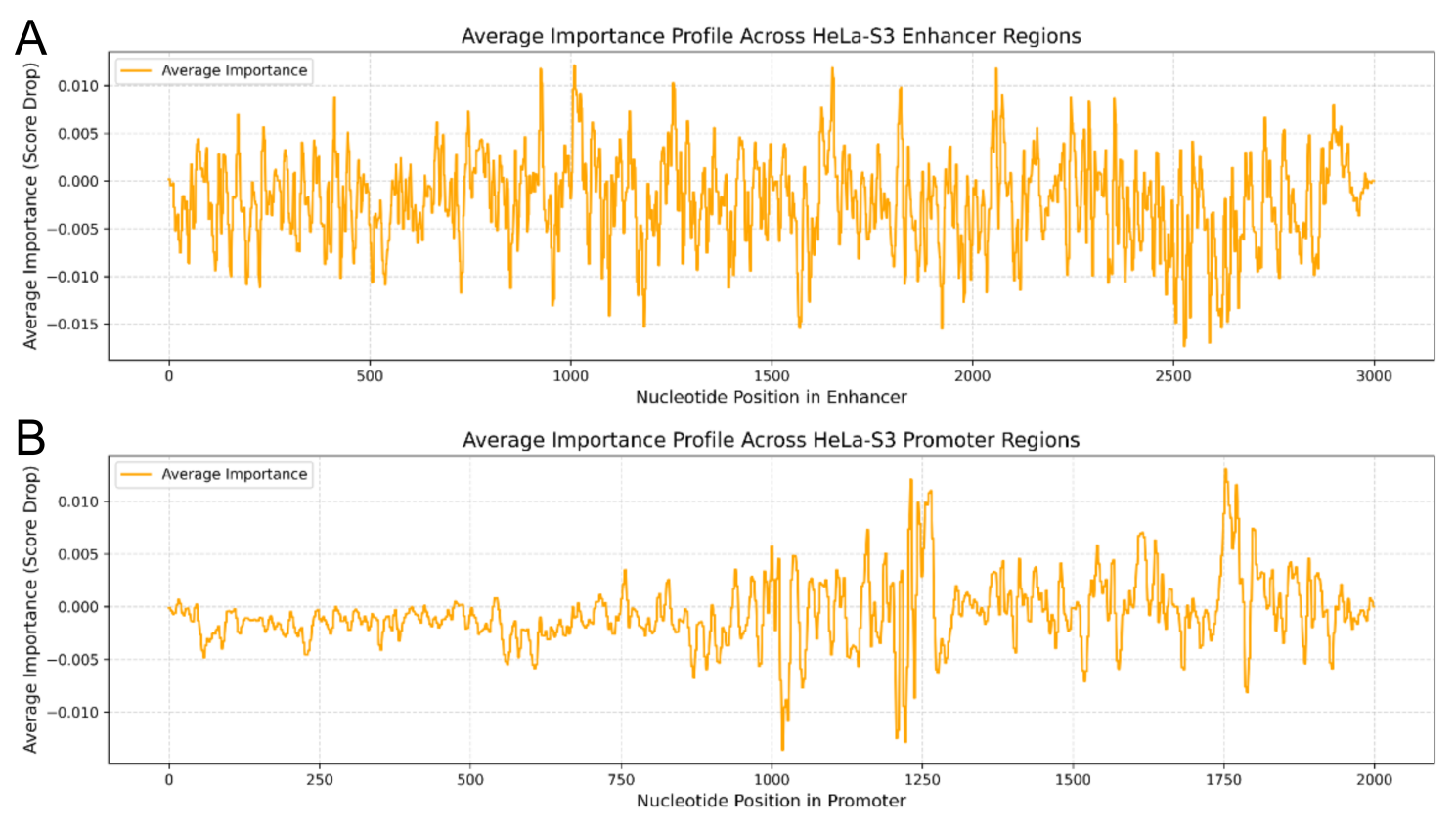
**

**Figure.2 Effect of perturbation at different positions of HeLa-S3 on the predicted scores** (A) Perturbation at HeLa-S3 enhancer (B) Perturbation at HeLa-S3 promoter. Where, the x-axis represents different positions on the DNA sequence, and the y-axis represents the score of the effect of perturbing the position on the predicted value, with higher peaks indicating that perturbing the position has a greater effect on the predicted score.

**
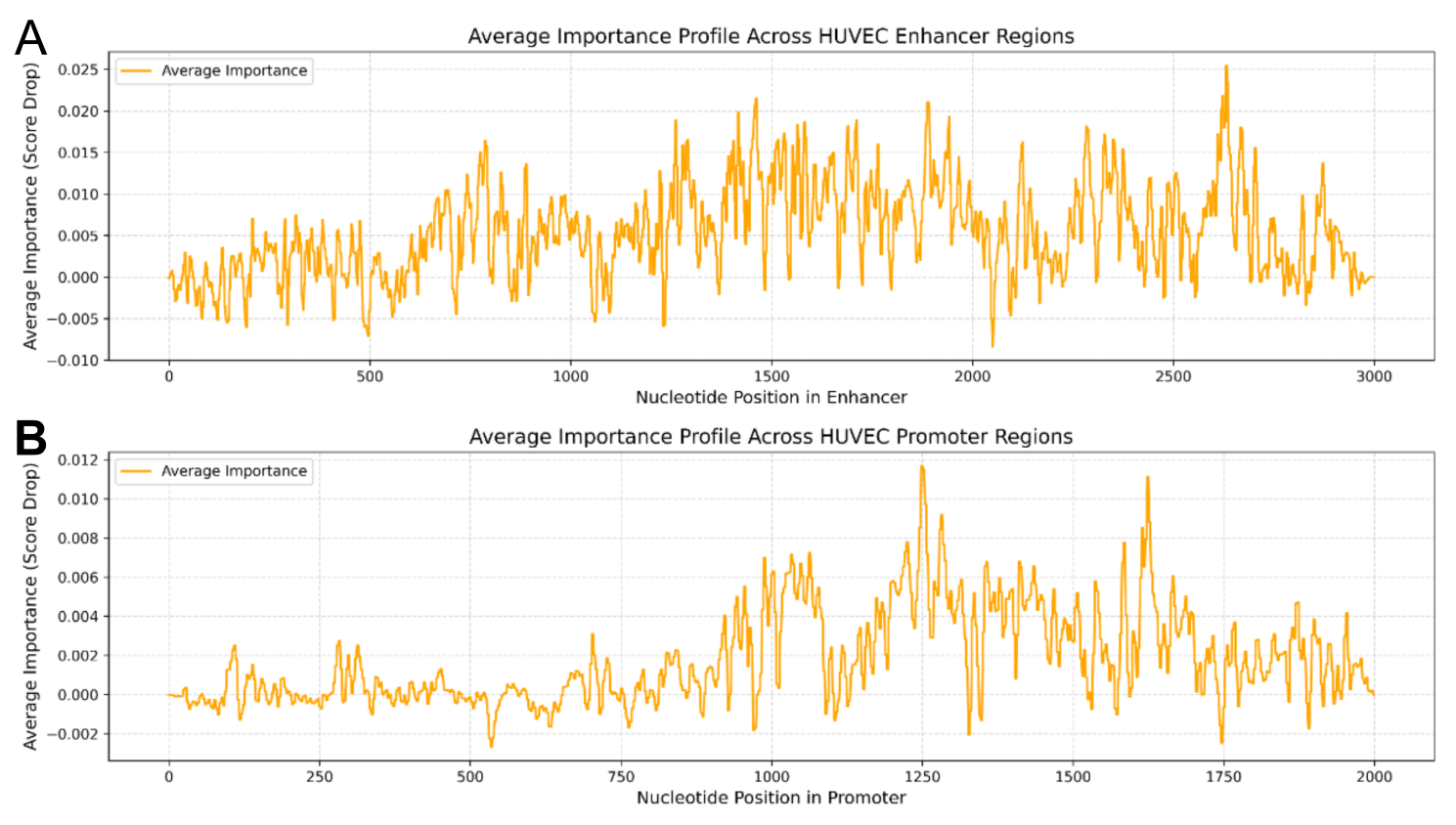
**

**Figure. 3 Effect of perturbation at different positions of HUVEC on the predicted scores.** (A) Perturbation at the HUVEC enhancer (B) Perturbation at the HUVEC promoter. where the x-axis represents different positions on the DNA sequence and the y-axis represents the effect of perturbing the position on the predicted value of the score, with higher peaks indicating that perturbing the position has a greater effect on the predicted score.

**
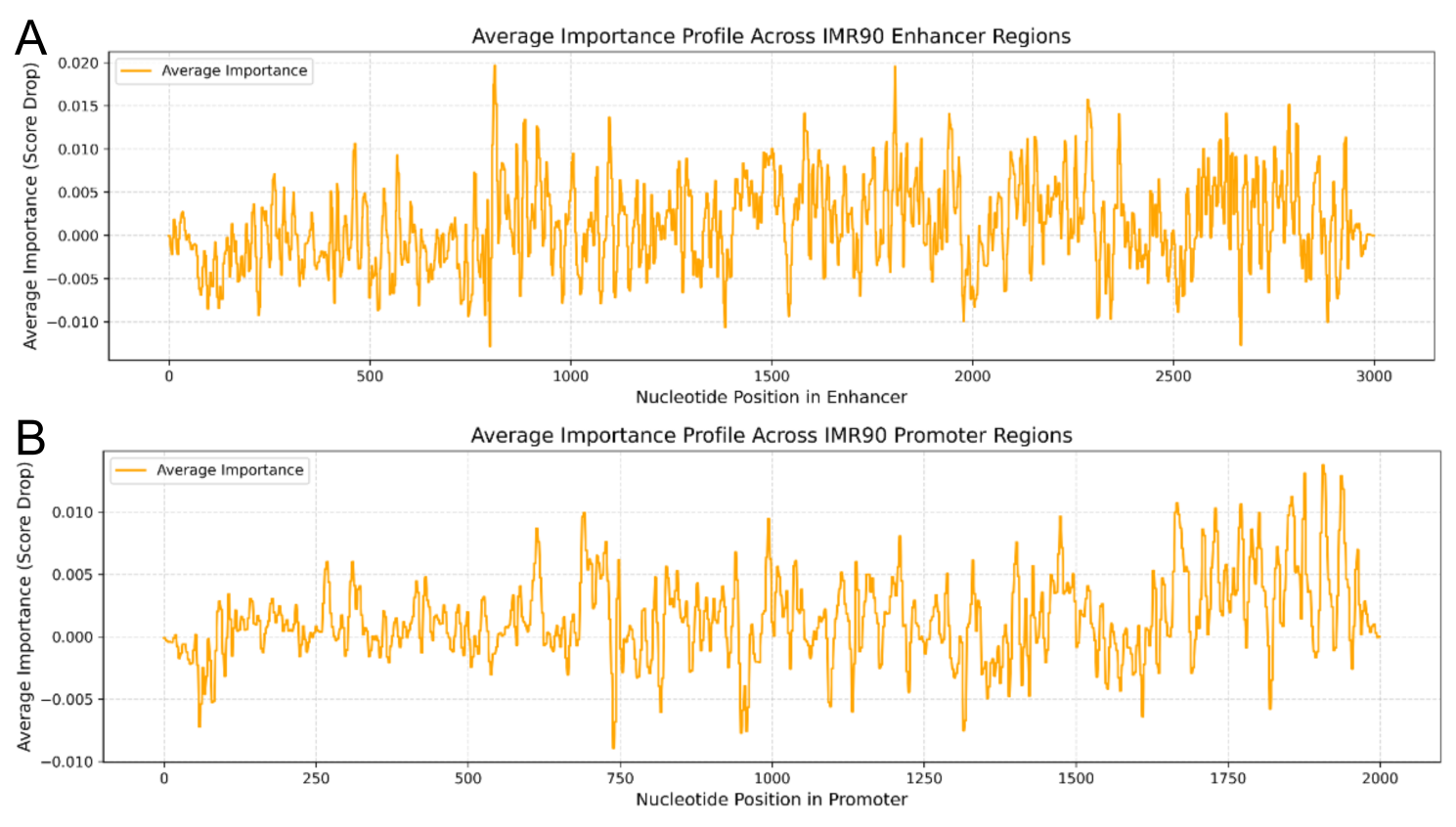
**

**Figure. 4 Effect of perturbation at different positions of IMR90 on the predicted scores.** (A) Perturbation at the IMR90 enhancer (B) Perturbation at the IMR90 promoter. where the x-axis represents different positions on the DNA sequence and the y-axis represents the effect score of perturbing the position on the predicted value, with higher peaks indicating that perturbing the position has a greater effect on the predicted score.

**
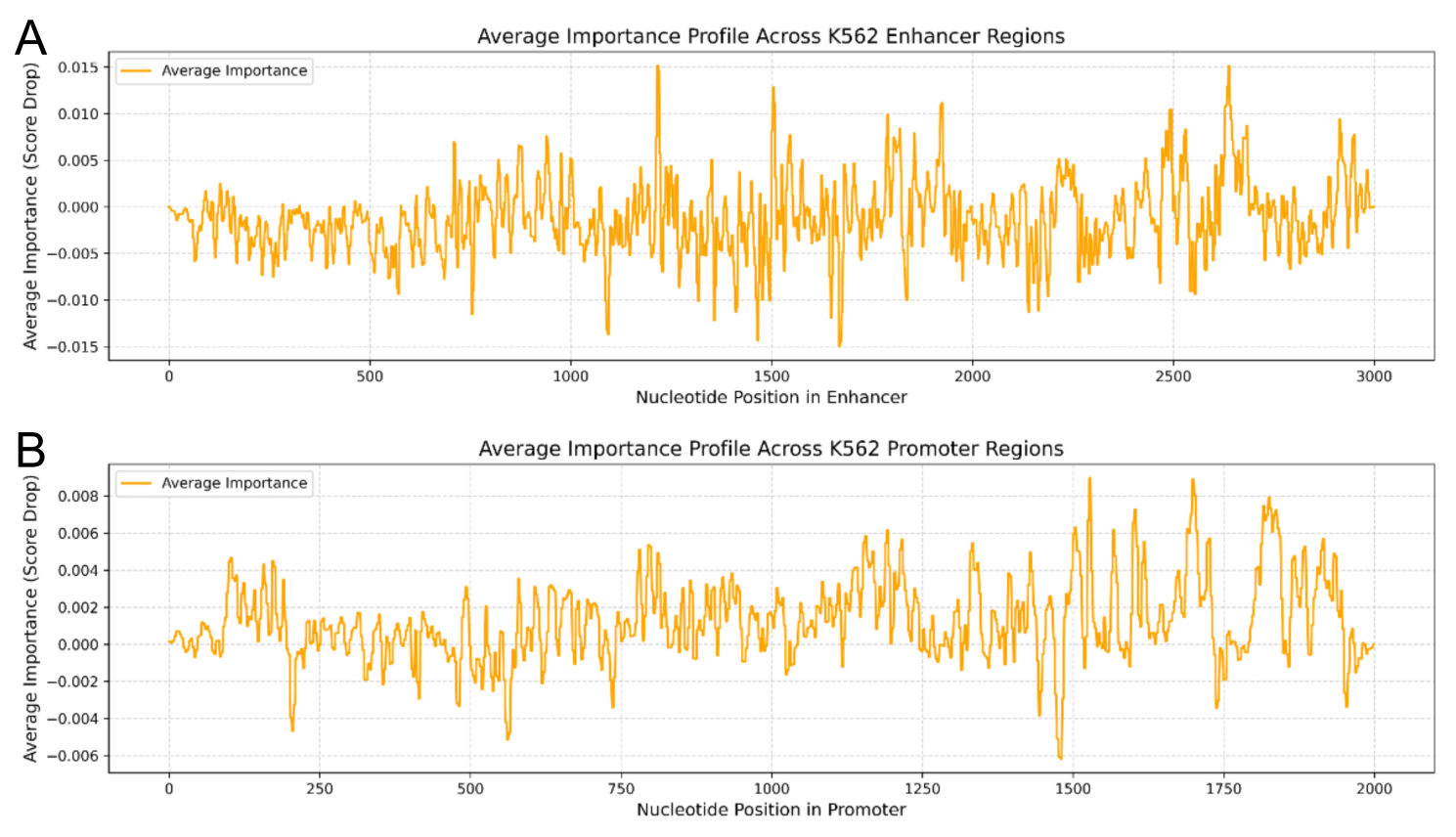
**

**Figure. 5 Effect of perturbation at different positions of K562 on the predicted scores.** (A) Perturbation at the K562 enhancer (B) Perturbation at the K562 promoter. where the x-axis represents different positions on the DNA sequence and the y-axis represents the effect of perturbing the position on the predicted value of the score, with higher peaks indicating that perturbing the position has a greater effect on the predicted score.

**
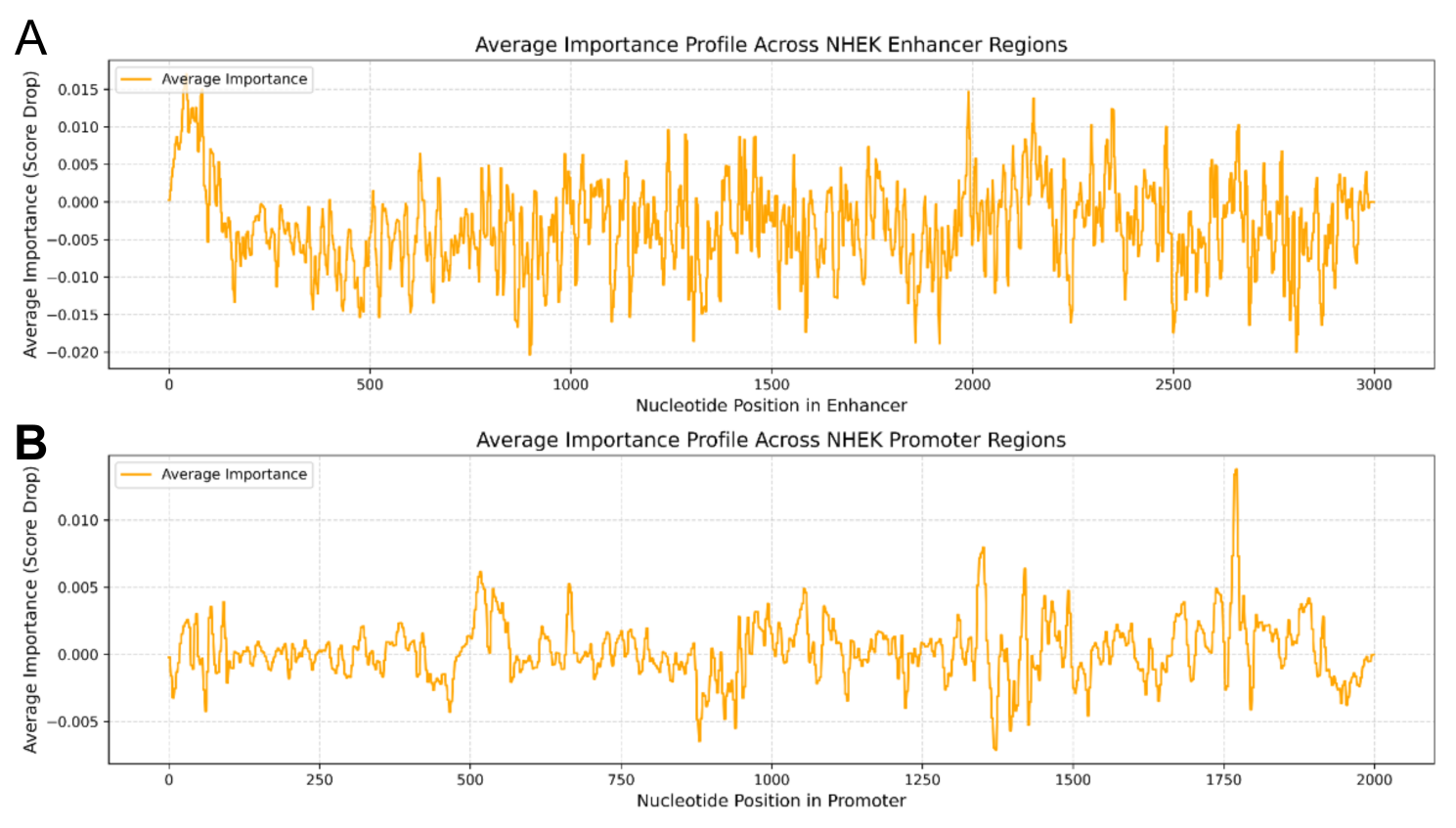
**

**Figure. 6 Effect of perturbation at different positions of NHEK on the predicted scores.** (A) Perturbation at the NHEK enhancer (B) Perturbation at the NHEK promoter. Where, the x-axis represents different positions on the DNA sequence, and the y-axis represents the effect score of perturbing the position on the predicted value, with higher peaks indicating that perturbing the position has a greater effect on the predicted score.

# Supplementary formulas

$$baseline\_score=f(en\_orig,pr\_orig)$$

$perturbed\_score=f(en\_input,pr\_input)$

$$score=baseline\_score-perturbed\_score$$

$${importance}_{i}=\frac{1}{n_{i}}\sum_{j=1}^{n_{i}} {score}_{j}$$

where $f(\cdot)$𝑓is the model's prediction function, $en\_orig$ and $pr\_orig$ are the input sequences of the original enhancer and promoter regions, $en\_input$ and $pr\_input$ are the perturbed enhancer and promoter sequences. $baseline\_score$ represents the baseline prediction score, $perturbed\_score$ represents the prediction score after perturbation, and $\mathrm{score}$ represents the significance score.$n_{i}$ denotes the number of sequences in the i-th cluster, $\mathrm{score}_{j}$ represents the significance score of the j-th sequence in the cluster, and $\mathrm{importance}_{i}$ is the importance score of the i-th cluster.
